# Amide Spectral Fingerprints are Hydrogen Bonding-Mediated

**DOI:** 10.1021/acs.jpclett.2c01277

**Published:** 2022-06-30

**Authors:** Sara Gómez, Cettina Bottari, Franco Egidi, Tommaso Giovannini, Barbara Rossi, Chiara Cappelli

**Affiliations:** † Scuola Normale Superiore, Classe di Scienze, Piazza dei Cavalieri 7, 56126, Pisa, Italy; ‡ Elettra Sincrotrone Trieste S.C.p.A., S. S. 14 Km 163.5 in Area Science Park, I-34149, Trieste, Italy; § Department of Physics, University of Trento, via Sommarive 14, I-38123 Povo, Trento, Italy

## Abstract

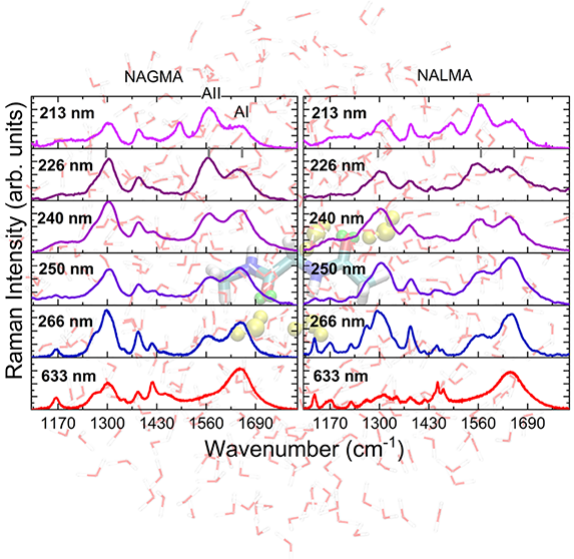

The origin of the peculiar amide spectral features of proteins in aqueous solution is investigated, by exploiting a combined theoretical and experimental approach to study UV Resonance Raman (RR) spectra of peptide molecular models, namely *N*-acetylglycine-*N*-methylamide (NAGMA) and *N*-acetylalanine-*N*-methylamide (NALMA). UVRR spectra are recorded by tuning Synchrotron Radiation at several excitation wavelengths and modeled by using a recently developed multiscale protocol based on a polarizable QM/MM approach. Thanks to the unparalleled agreement between theory and experiment, we demonstrate that specific hydrogen bond interactions, which dominate hydration dynamics around these solutes, play a crucial role in the selective enhancement of amide signals. These results further argue the capability of vibrational spectroscopy methods as valuable tools for refined structural analysis of peptides and proteins in aqueous solution.

Amide bands are considered sensitive probes of the secondary structures of proteins and peptides enabling prediction that provides a significant advance in the knowledge of protein activity and function. For this reason, vibrational spectroscopy experiments such as Infrared and Raman are well-established methods to identify and quantify distinct secondary structure motifs of proteins and polypeptides through exploration of the Amide fingerprint region.

The proper interpretation of ever more accurate experimental measurements makes the availability of reference studies analyzing the deep nature and physical origin of the spectroscopic response down to the atomistic detail highly desirable. *N*-Acetyl-glycine-methylamide (NAGMA) and *N*-acetyl-leucine-methylamide (NALMA) (see Figure 1) can be employed as minimal prototypes to model certain protein properties and behaviors.^1−12^ Compared to simple unmodified amino acids, both their C and N termini are modified to model the peptide bonding, while maintaining a small size and conformational flexibility; therefore, they are more suitable as “peptide models” than single amino acids, which do not exhibit the chemical heterogeneity and interactions that characterize a protein backbone. Being minimal models for larger molecular structures, they allow for extensive and highly detailed investigations into their physicochemical properties, both intrinsic and relating to their molecular environment, as well as the details of their spectroscopic properties,^1,13−22^ which is particularly crucial as advanced spectroscopic techniques are then applied to more complex biological polymers.

**Figure 1:**
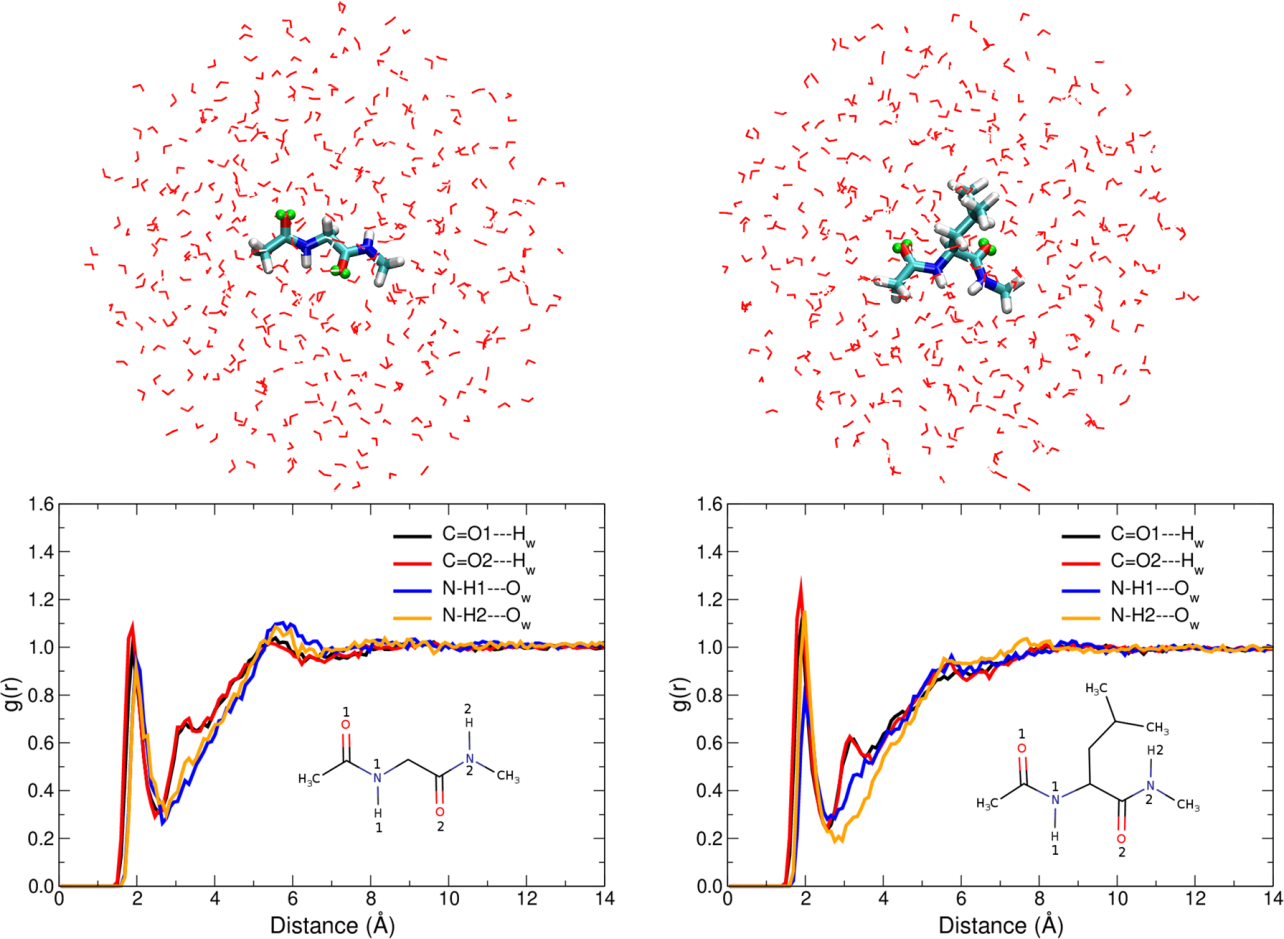
Top: QM/FQ representation of NAGMA (left) and NALMA (right) in aqueous solution. Virtual sites (VS) in the C=O groups are depicted in green. VS are interaction sites constructed to improve the description of the hydrogen bonding. Bottom: Radial Distribution Functions (RDFs) for the intermolecular O···H interactions in solvated dipeptides.

The simplicity of the molecular models, however, hides the complexity that exists, as these systems are dissolved in aqueous solution, i.e. their physiological environment. Any attempt at modeling these peptides must therefore focus on the nature of solvation and its effects upon the properties of the systems. Indeed, the investigation of the hydration dynamics at hydrophilic and hydrophobic biomolecular sites of simple peptides has paved the way to appropriate models for understanding the dynamics of the first hydration shell of proteins.^1,14,23^

The unique combination of the capabilities of UV Resonance Raman (UVRR) spectroscopy (enhanced detection limit, high selectivity of specific chemical groups, no interfering signal of water solvent in the amide fingerprint spectral region)^24−26^ and the features of these small peptides result in local molecular probes for focusing on hydrogen bond (HB) rearrangements at specific sites of peptides, even under high diluted conditions. In this regard, it has already been shown^22^ that, due to the advantages of Synchrotron Radiation (SR)-based UVRR, the fine-tuning of the excitation wavelength allows the experimenter to select the best working conditions that ensure one can reliably detect the spectral changes of amide signals, as a function of peptide concentration and temperature.^27,28^ However, there are still some experimental features that are not fully understood. First, as also demonstrated in this work, a selective enhancement of the Amide II (AII) mode is experimentally observed at the shortest excitation wavelengths for both NAGMA and NALMA in aqueous solution and not for their microcrystalline form. *Selectivity* in this context means that excitation of π → π* transitions of the amide peptide bonds, lead to strong UVRR enhancement of Raman signals associated with the vibrations that have large components of C–N stretching, while smaller enhancement occurs for vibrations with strong C=O stretching. Second, Amide I (AI) and AII shapes and positions change going from microcrystalline/hydrated powders to solutions of both peptides. Third, in the measured spectra a dependence of wavenumbers of amide bands on concentration is reported. In this work, we explain the physical origin of the three aforementioned experimental findings, by interplaying experiments and simulations.

Often, simulations are unable to reproduce experimental findings of (Resonance) Raman spectra due to the intrinsic limitations in standard solvation models.^29^ Solvation affects molecular response properties both directly and indirectly by altering a molecule’s conformational landscape. The effects of solvation are commonly estimated either by implicit, continuum approaches or by simulation studies (MonteCarlo or Molecular Dynamics (MD)) in which the solvent molecules are explicitly considered in both the sampling and the calculation of the properties.^30−34^ Recently, some of the present authors have designed a multiscale computational protocol, combining MD and the Quantum Mechanics/Fluctuating Charges (QM/FQ) approach, a polarizable embedding model that keeps a fully atomistic representation of the solvent and provides accurate excitation energies and RR spectra of various systems, including small amides.^35,36^

Disentangling the role of each type of interaction in the generation of the UVRR spectra of these systems is a necessary first step if one hopes to fully rationalize and explain the spectroscopic behavior of peptides in aqueous solution, and due to the aforementioned complexities, this result can only be achieved by combining the highest possible level of techniques, both theoretical and experimental. To tackle these problems we therefore used the QM/FQ model in combination with SR-UVRR experiments at different excitation wavelengths and interpret, at the molecular level, the selective enhancement of the Amide II band experimentally detected at the shortest wavelengths. To this end, we performed extensive simulations using a hierarchy of solvation approaches on NAGMA and NALMA (see Computational Methods in the Supporting Information (SI)) and investigated the role of hydrogen bonding in the spectroscopic behavior of these systems, by comparing simulated results^37,38^ with the multiwavelengths experimental measurements made possible by the fine energy tunability of a SR source, which affords a much greater degree of flexibility compared to standard experimental setups, where the excitation radiation is provided by energy-fixed laser sources. Experimental procedure and terminology are part of the SI.

We began by examining the performance of QM/FQ, coupled to MD sampling, to describe UV–vis, Raman, and RR spectra of the two peptide systems (see Figure 1 for a picture of the representative systems used in the simulations). Furthermore, we evaluated gas phase, Polarizable Continuum Model (PCM), and cluster calculations as summarized in Table 1. All studied systems are depicted in Table S1 in the SI; their structural parameters are listed in Tables S2 and S3.

**Table 1:** Computed Vertical Excitation Wavelengths (in nm) for NAGMA and NALMA in Different Environments, Calculated at the B3LYP/6-311++G(*d*, *p*) Level of Theory^c^

		Absorption maxima (nm)	
Motif	Environment	NAGMA	NALMA
Monomer *C5*	Gas phase	200.4, 180.5	190.1
Monomer *C7*	Gas phase	196.6	196.0
Monomer β_2_	Gas phase	194.6	200.2
Monomer *C5*	PCM	178.9	184.1
Monomer *C7*	PCM	182.6	187.9
Monomer β_2_	PCM	184.5	189.0
Monomer + 4W	PCM	181.7	183.8
Solution (366:1)	QM/MM NP	174.0	177.3
Solution (366:1)	QM/FQ^a^	175.2	178.5
Solution (366:1)	QM/FQ^b^	175.3	177.5
Solution (366:1)	QM/QM_*w*_/FQ	178.3	181.5
Dimer	Gas phase	197.0	199.5
Dimer solvated	Gas phase	190.9	193.9

a FQ parametrization from ref (42).

b FQ parametrization from ref (43).

c NP stands for the Non-polarizable TIP3P.^39^ Numbers in parentheses indicate the ratio water molecules: peptide molecules. In amides/small peptides, the first allowed electronic transition is experimentally reported to occur at ca. 190 nm^40,41^

Due to their conformational flexibility, NAGMA and NALMA monomers may feature intramolecular HBs. Consistent with previous reports,^8,10,12^ we found that in the gas phase the stable conformers are located mainly in *C5* (β_*L*_) and *C7* (γ) regions, while solvent effects increase the prevalence of minima in the α and β_2_ (δ_*L*_) regions. Ramachandran maps in Figure S2 in the SI indicate that β_2_ is the most representative conformer sampled by MD runs.

In dilute aqueous solution intramolecular hydrogen bonds compete with intermolecular interactions between solute and solvent, with the latter becoming dominant, as supported by the intra- and intermolecular radial distribution functions (RDFs) for the O···H contacts and by the HB strength estimated using Natural Bond Orbitals (NBO)^44^ (Figures S3 and S4 in the SI). Intermolecular RDFs are shown in Figure 1. For both molecules, the C=O and N–H groups of the backbone are involved in the same number of HBs (2, 1 each). Building upon this observation, we have saturated all potential HB sites using 4 water molecules to build what is generally called a “supermolecule” (see Table S1 in the SI).

Despite sharing some features, the maxima in the RDFs are always located at slightly shorter distances for NAGMA, confirming that the strength of the solute–solvent interactions is higher for NAGMA than for NALMA, due to the hydrophobic residue in the latter. This finding is consistent with the detected wavenumber shift between AI and AII signals in the UVRR spectra of NAGMA and NALMA that reflects a different strength of peptide–solvent interactions for the two peptides.^22^

Considering that strong UVRR intensities are to be expected when properly tuning excitation wavelengths, we calculated the UV–vis spectra of both molecules in the different environments considered in this study. Table 1 lists vertical excitation energies in each case. The bands in the electronic absorption spectra are very similar in appearance (Figure S5 in the SI), with the aqueous solution bands being blue-shifted by up to 20 nm relative to *C7*, the most stable conformer in the gas phase. In contrast, and regardless of the solvent representation, slight differences (5 nm at most) are noted in the case of the aqueous solution. We assign the strong electronic absorption band computed at ∼180 nm (190 nm is the experimental report) for both systems as the π → π* transition based on prior studies^40^ and the orbitals involved (Figure S6 in the SI). Thereupon, excitation within this absorption band will give rise to enhancements of the peptide bond vibrations.

Raman (Far From Resonance, FFR) and UVRR spectra of solvated NAGMA and NALMA are displayed in Figure 2. At first glance, all the typical vibrational features can be recognized in both the measured and computed spectra, namely, (*i*) the presence of the Amide I (AI) band with a Raman signal around 1650 cm^–1^ which is mainly associated with the stretching vibration of the C=O of the amide linkage in the peptide backbone; (*ii*) the Raman signal at ∼1560 cm^–1^, assigned to the Amide II (AII) and resulting by the out-of-phase combination of N–H bending and C–N stretching movements of the groups in the amide linkage; and (*iii*) the Amide III (AIII) band, whose vibrational mode is mostly due to the in-phase combination of N–H bending and C–N stretching and which appears around 1260 cm^–1^. Notice that in the FFR visible spectra of peptides the Amide II mode is completely absent (as expected), while in the UVRR spectra this band clearly appears even in preresonance conditions (excitation wavelength at 266 nm). Normal modes are drawn in Figures S7 and S8 in the SI.

**Figure 2:**
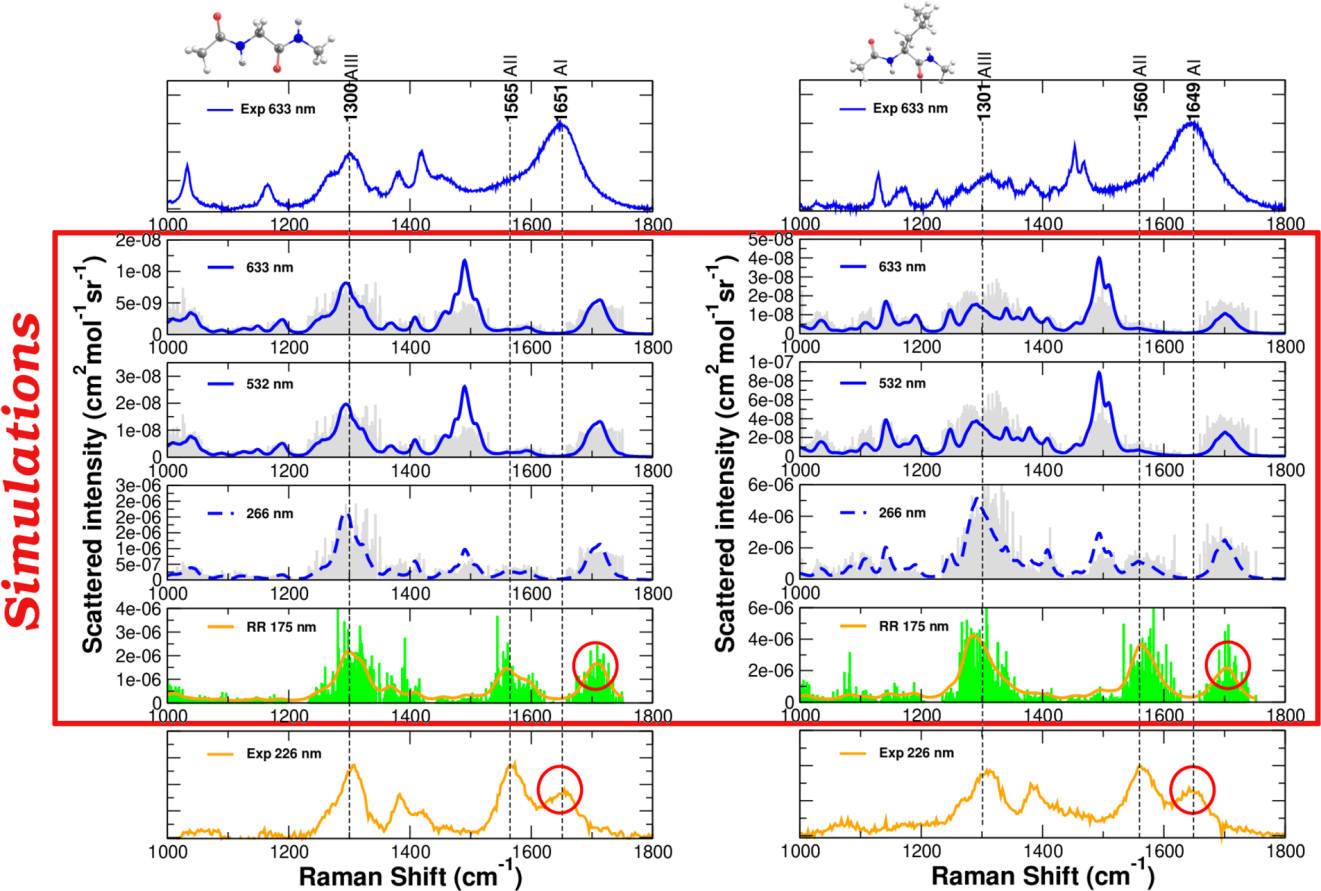
Raman (blue) and Resonance Raman (orange) spectra of NAGMA and NALMA, left and right panels, respectively. Experimental spectra were measured at room temperature in aqueous solution at a concentration which corresponds to 366 molecules of water for each molecule of peptide. QM/FQ results for spontaneous (Far From Resonance) and Resonance Raman spectra were broadened using Lorentzian functions with an fwhm of 8 and 20 cm^–1^ respectively. RR intensities were calculated with a damping factor of 200 cm^–1^. Sticks in the simulated spectra are also included. The dashed blue curve indicates a preresonance condition.

The above results show that the mutually polarizable QM/FQ approach describes well Raman and RR spectra of the two systems, while fairly preserving positions and relative intensities, unlike results in PCM or using cluster/supermolecule approaches (see Figure S9 in the SI). However, it can be noted that when the FQ parameters taken from ref (42) are utilized, the accuracy in predicting AI band position is not entirely satisfactory (see red circles in Figure 2). One direction of improvement could be the usage of a different parametrization, where electrostatics and polarization effects are more accurately accounted for in the QM Hamiltonian.^43^ In fact, when FQ^*b*^ parameters are used, AI and AII bands approach each other, thus moving computed results toward experimental data (Figure S10 in the SI). Detailed analysis of the normal modes obtained with the parameters of ref (43) suggests that AI and AII modes are coupled.

From the UVRR spectra in Figure 2, AI, AII, and AIII are the signals found to be particularly affected by the resonance enhancement, making their simultaneous measurement the ideal experimental target to be used to directly determine protein secondary structure.^45^ Nevertheless, the experimental scanning along the excitation wavelength reveals a selective enhancement of the AII mode at the shortest wavelengths, as shown in Figure 3a. This feature has been already pointed out in literature for peptides and proteins^22,46,47^ and is also relevant here, even though it is slightly more intense for NAGMA than for NALMA (compare for example spectra at 226 and 213 nm in Figure 3a) as can be seen in the ratio of the areas in Figure 3b. Interestingly, such an effect is observed only for solutions of peptides and not for the microcrystalline form of the molecules (see Figure S11 in the SI). This latter experimental evidence suggests a crucial role played by the hydration shell around the peptides in determining the spectral features of Amide Raman signals.

**Figure 3:**
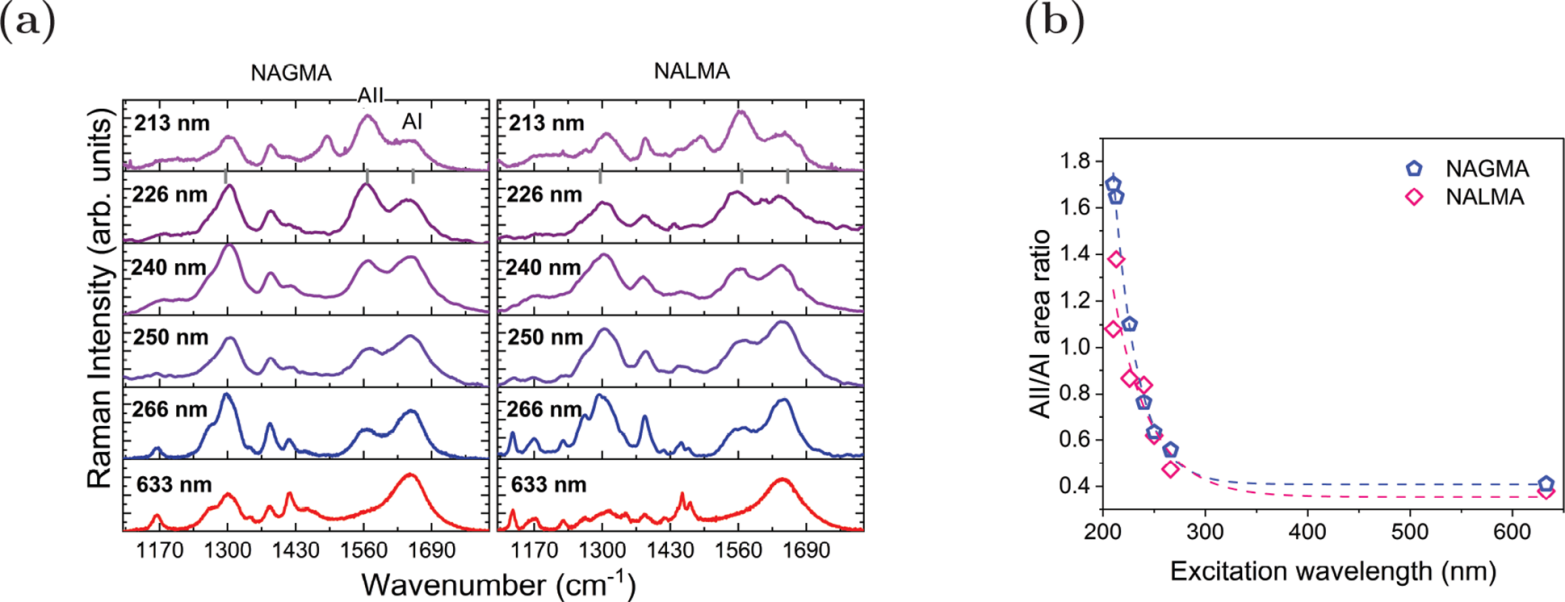
Experimental results for NAGMA and NALMA dissolved in water at a concentration corresponding to 366 molecules of water for each molecule of peptide (a) FFR and UVRR spectra collected using different excitation wavelengths ranging from visible to deep UV energies. (b) Estimated ratio of the areas of amide modes AII/AI as a function of the excitation wavelength.

An explanation for the selective AII intensity increase has been proposed^48^ in terms of the stabilization of the ground state dipolar resonance structure −O–C=NH_2_^+^ that becomes more favored in aqueous solution with respect to the O=C–NH_2_ resonance form that lacks charge separation. Thus, the formation of HBs at the amide and carbonyl sites leads to a contraction of the C–N distance (see Table S2 in the SI) and the vibrations having significant contributions from the C–N stretching mode, namely, amide I, II, and III, will have appreciable intensity enhancements in the Raman lines. Our calculations of resonance structures using Natural Resonance Theory (NRT)^49^ indicate that 3 out of 4 main NAGMA resonance hybrids favor the dipolar form, namely, negative charges in the O atoms and positive charges on the N atoms (Figure S12 in the SI), and more importantly, the percentage values of all the hybrids bearing formal charges increase in solution. Indeed, when explicit water molecules are considered, dipolar structures become the leading resonance hybrids (Table S4 in the SI). The special preference for enhancing AII when excitation wavelengths approach the maxima in the absorption spectra might be further analyzed based on the orbitals involved in the in-plane π → π* transition. Computing 15 TD-DFT excited states on dipeptides structures revealed that the excited states with the highest oscillator strengths have an important charge transfer contribution, predominantly from HOMO and HOMO–1 to a wide assortment of virtual orbitals. For glycine and leucine dipeptides in solution, such states have larger contributions from the C–N regions of the molecules (Figure S6 in the SI). In addition, it is evident from inspection of Figure S6 (in the SI) that the orbitals involved not only belong to the solute but also involve the nearest water molecules, thus implying that charge transfer between peptides and solvent is an active component of molecular orbitals contributing to the 190 nm band. In view of the above findings, modeling the selective enhancement would require the inclusion of a few solvent molecules in the QM portion of the system.

Computed Resonance Raman Excitation Profiles (RREPs) are shown in the SI, Figures S13–S17 and S18–S22, for NAGMA and NALMA, respectively. The ratio between the areas of AI and AII in the simulated spectra using excitation wavelengths ranging from 633 to 166 nm is plotted in Figure S23 in the SI. Although QM/FQ with the two sets of parameters gives reasonable descriptions of the spectra, both parametrizations produce always an AI band with higher intensity and area than AII, leading to discrepancies when compared to the experiments. Conversely, it seems to be an important trend for the resonance enhancement of AII in the modeled RREPs of solvated NAGMA in the supermolecule case.

According to our molecular dynamics simulations and those reported by Boopathi and Kolandaive,^6^ an average of two and three water molecules form persistent interactions with NALMA and NAGMA dipeptides, respectively. Also, it is well-known that the pattern of the relative intensities of the amide bands is determined by the properties in the electronic excited states for the various conformations.^45,47^ Hence, to gain a deeper insight into the effect of these solvent molecules on the selective intensity alteration, we have investigated the intensity dependence on the exciting frequency of the AII by quantum-mechanically treating all water molecules that fulfill at least one of the following geometric criteria:  Å or  Å, hereafter noted as the QM/QM_*w*_/FQ approach (details in Table S5 in the SI). This is graphically depicted in Figure 4b for NAGMA. A better reproduction of the experimental trend shown in Figure 3a was found when explicit water molecules are part of the QM layer, as seen in Figure 4b for two selected wavelengths. Furthermore, there is excellent agreement between the ratio of the areas, dashed curves in Figure 4c, and its experimental counterpart, Figure 3b. This constitutes an important piece of evidence of the role of hydrogen bonding and, specifically, its quantum-mechanical covalent component, in the intensity enhancement because just with solvent molecules linked to the C=O and N–H groups, the distances within the peptide are properly modified. Moreover, the orbitals of the transition are more concentrated in the C–N regions of the molecules, which ultimately triggers the enhancement. Regarding the NALMA case, the selective enhancement is seen in the QM/QM_*w*_/FQ solvation model only. So, we deduce that there is a similar solvation behavior for the two peptides and that the hydrophilic part of the two molecules dominates the spectral properties, in addition to the solvation dynamics explained in ref (22).

**Figure 4:**
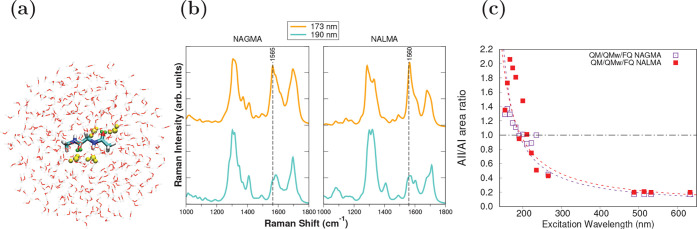
Computational results obtained with the QM/QM_*w*_/FQ approach applied to NAGMA and NALMA in aqueous solution. (a) Representative structure, where selected water molecules (in yellow) are part of the QM portion. (b) Comparison between UVRR spectra simulated at 173 and 190 nm. (c) Computed ratio of the areas of the amide modes AII/AI as a function of excitation wavelength.

Next, the different shapes and positions for AI and AII are studied by focusing on the UVRR spectra of the microcrystalline forms (Figure S11 in the SI), hydrated powders (Figure S24 in the SI), and solvated (Figure 2) samples. Explicitly in the amides region, some clear distinctions are evident: on one hand, a significant difference in the shape of AI for the two peptides, with probably two subcomponents with slight changes in frequency and relative intensity. Such an experimental splitting of the AI band (NAGMA case) is also recovered by the calculated spectra and explained by having a look at the normal modes, where two sets of C=O oscillators (see modes 21 and 22) with slightly different strengths can be identified. In contrast, they are more compactly gathered in the NALMA case. This observation is in line with the two types of C=O···H interactions found in the hydration patterns (Figure S3 in the SI), due to the arrangements of the molecules’ backbone. On the other hand, it is also observed from Figure S24 in the SI that the position of Amide II (AII) has a red shift of about 5 cm^–1^ in the NALMA case. We recall here that the effect of the hydrophobic portion of NALMA is the lengthening of the N–H···O_*w*_ distances with respect to NAGMA, thus affecting the N–H bending associated with that band.

Finally, in order to rationalize the concentration-dependence of wavenumber positions, it is worth mentioning that at high dilution conditions, like those in our simulations, solute–solute interactions are weak (see NBO interaction energies, Figure S4 in the SI) and then the C=O is expected to be more involved in HBs with solvent molecules. In particular, it has been seen^22^ that for concentrations ≤50 mg/mL of both molecules in water (i.e., 144 molecules of water for each molecule of peptide), the position of the AI band becomes strongly dependent on concentration (severe red shift upon the increment of water content). Based upon the NRT results (Figure S12 and Table S4 in the SI), we argue that in aqueous solution, with the stabilization of the dipolar resonance structures, the double bond is more localized between C and N, leading to a shortening of the C–N bond and in turn to a lengthening of the C=O bond. Therefore, the force constant of a bond having a stronger single character compared to the resonance form that does not have a charge separation induces a decrease in the oscillation frequency for the C=O stretching and so a red shift of AI. The same explanation would apply for the concentration-dependent trend of AII in concentrations >50 mg/mL (144:1 water/peptide) where probably the solute–solute interactions become important. Aggregation propensities of these peptides in solution have been examined before in the literature.^4^ Here, we explore solute···solute interactions through an approximate treatment, namely, the dimeric and solvated dimeric forms of NAGMA and NALMA. Their modeled RR can be found in Figure S25 in the SI. While in the UVRR spectra of concentrated dipeptide solutions it is reported^22^ that there are no significant changes in the AI frequency position, AII, arising from the combination of C–N stretching and N–H bending motions, experiences a slightly monotonic blue shift, likely due to the strengthening of the C–N bond, which causes an increase in the force constant and, in turn, in the oscillation frequency. To verify the reliability of these arguments, we carefully checked the distances of the C=O and C–N in Tables S2 and S3 in the SI and concluded that, in all our representations of the solvent, the C=O and C–N bond lengths are actually longer and shorter, respectively, compared to the isolated cases. Consequently, it is again verified that the solvent does play a role in determining the positions, shapes, and intensities of the RR bands, as also pointed out by some authors.^28,50−52^

In summary, the interplay of computational and experimental data highlights two important observations: (*i*) The selective enhancement of the amides signals is hydrogen bonding-induced because it is intimately linked to the effect that water molecules exert on the C=O and N–H, C–N vibrations. We demonstrated that the inclusion of explicit water molecules concentrates the orbitals involved in the charge transfer in the C–N zones, which ultimately leads to the strong UVRR enhancement of vibrations that have large components of C–N stretching, particularly the AII signal. Thus, quantum effects must be present in any modeling of the solute–solvent interactions of RR spectroscopy for such systems. The unprecedented accordance found between the theoretical calculation and experimentally collected Raman and Resonance Raman spectra further testifies the reliability of our model. (*ii*) Due to the constant movement of the solute and its surrounding water molecules, a single snapshot (or cluster composed of the solute and some surrounding water molecules) is not representative of the dynamical nature of the system and can lead to heavily biased results if taken to be representative for the ensemble, which is more correctly modeled through an explicit average over a large set of structures. Through our investigation we put forward an explanation at the molecular level of the origin of the selective enhancements, emphasizing the crucial role of the backbone conformations and the dynamics of their surrounding waters. These results provide an important starting point to calibrate wavenumbers and intensities of the experimental Amide signals for the quantitative determination of structural parameters of protein and peptide in solutions. A key requirement of computational approaches to be truly useful and complementary for experimental measurements is the ability to properly describe and reproduce spectral features, and not just the energetics of a system. We have shown that the QM/QM_*w*_/FQ fulfills this by promisingly going beyond standard methodologies based on more crude approximations and is, therefore, expected to open up new possibilities for novel applications in a truly synergistic partnership with advanced experimental techniques applied to biologically relevant samples, as well as shed new light regarding the details of physicochemical phenomena that characterize the functioning of life. In this respect, our study could be also extended to Raman Optical Activity (ROA) spectroscopy, which, due to its sensitivity to chirality, constitutes an alternative to Raman/Resonance Raman when examining the structure and behavior of peptides, proteins, and biomolecules.^53−56^

## Supporting Information Available

Description of the experimental and computational methods, structural analysis, hydration patterns, measured and calculated spectra, Resonance Raman Excitation profiles, normal modes. (PDF)
