# Supplementary material for: Amide Spectral Fingerprints are Hydrogen Bonding-Mediated
Source: J Phys Chem Lett. 2022 Jun 30;13(26):6200–7. doi: 10.1021/acs.jpclett.2c01277 (PMC9272440; doi:10.1021/acs.jpclett.2c01277)
Supplement: Supplementary file 1 — jz2c01277_si_001.pdf [file jz2c01277_si_001.pdf]

Amide Spectral Fingerprints are Hydrogen Bonding-Mediated

Sara Gómez,[†,*] Cettina Bottari,[‡] Franco Egidi,[†,¶] Tommaso Giovannini,[†] Barbara Rossi,[‡] and Chiara Cappelli,[†,*]

[†]*Scuola Normale Superiore, Classe di Scienze, Piazza dei Cavalieri 7, 56126, Pisa, Italy*
[‡]*Elettra Sincrotrone Trieste S.C.p.A., S. S. 14 Km 163.5 in Area Science Park, I-34149, Trieste, Italy and Department of Physics, University of Trento, via Sommarive 14, I-38123 Povo, Trento, Italy*
[¶]*Software for Chemistry & Materials BV, De Boelelaan 1083, 1081 HV Amsterdam, The Netherlands*

Correspondence: sara.gomezmaya@sns.it; chiara.cappelli@sns.it

Supporting Information

Contents

1 Experimental procedure **S3**
1.1 Samples . . . S3
1.2 Out of resonance Raman measurements . . . S3
1.3 SR-UVRR measurements . . . S3

2 Computational Methods **S4**
2.1 Conformational studies . . . S4
2.2 MD simulations . . . S4
2.3 Calculations of the Spectra . . . S5

3 Structural analysis **S7**
3.1 Ramachandran plots . . . S11
3.2 Hydration patterns . . . S12
3.2.1 RDFs . . . S12
3.2.2 NBO interactions . . . S15

4 Spectra and further analysis **S16**
4.1 Computed UV/Vis absorption spectra . . . S16

4.2 Normal modes S18
4.3 Simulated UVRR spectra for NAGMA and NALMA in aqueous solution . . S20
4.4 Experimental UVRR spectra of the peptides in their microcrystalline form . S23
4.5 Natural Resonance Theory (NRT) Analysis S24
4.6 Simulated RR Excitation Profiles S24
4.6.1 NAGMA S25
4.6.2 NALMA S30
4.7 Analysis of excitation wavelength and amide band intensities S35
4.8 Details about the QM/QM$_w$/FQ approach S36
4.9 Experimental UVRR spectra of hydrated powders S37
4.10 Simulated UVRR spectra in the gas phase for monomers and aggregates . . S38

1 Experimental procedure

1.1 Samples

The dipeptides N-acetyl-leucine-methylamide (NALMA) and N-acetyl-glycine-methylamide (NAGMA) were purchased from Bachem, and used without further purification. They appear both as micro-crystalline powders. No relevant contamination by water was found in the crystalline peptide powders, as deducted by the absence in the Raman spectra of any signal attributable to the intense OH stretching band of water. The aqueous solutions of dipeptides were prepared by dissolving NAGMA or NALMA in doubly distilled deionized water in order to reach the desired concentrations, typically corresponding to a molar ratio of 1:336 peptide:H_2O. All the solutions were freshly prepared before the measurements. Hydrated powders of NAGMA and NALMA were prepared by adding to the dry peptides a controlled amount of water up to reach the molar ratio of about 1:10 peptide:H_2O. The exact amount of added hydration water was determined by weight of the samples.

1.2 Out of resonance Raman measurements

Visible Raman spectra were collected on the microcrystalline forms and on the aqueous solutions of dipeptides by means of Raman setup (Horiba-JobinYvon, LabRam Aramis) in backscattering geometry and using the exciting radiation at 632.8 nm provided by a He-Ne laser and at 532 nm provided by a solid state laser. The spectral resolution was set at about 1 cm^{-1}.

1.3 SR-UVRR measurements

Synchrotron-based UV Resonance Raman (SR-UVRR) measurements were collected at the BL10.2-IUVS beamline of Elettra-Sincrotrone Trieste (Italy) using the experimental setup described in detail in ref 1. The UVRR spectra were acquired at different excitation wavelengths in the deep UV range provided by the emission of synchrotron radiation (SR). The energy of excitation was set by regulating the undulator gap aperture and using a Czerny-Turner monochromator (Acton SP2750, focal length 750 mm, Princeton Instruments, Acton, MA, USA) equipped with a holographic grating with 3600 groves/mm for monochromatizing the incoming SR. Raman signal was collected in back-scattered geometry, by a single pass of a Czerny-Turner spectrometer of 750 mm focal length and equipped with holographic gratings at 1800g/mm and 3600 g/mm. The resolution was set at different values, depending on the excitation wavelength (e.g. 2.8 cm-1/pixel at 210 nm and 1.8 cm-1/pixel at 266 nm). The calibration of the spectrometer was standardized using cyclohexane (spectroscopic grade, Sigma Aldrich). The final radiation power on the samples was kept between a few up to tens of μW. Any possible photo-damage effect due to prolonged exposure of the sample to UV radiation was avoided by continuously spinning the sample cell during the measurements.

2 Computational Methods

2.1 Conformational studies

Potential NAGMA and NALMA conformers were initially searched by 2-dimensional potential energy surfaces (PES), built by scanning its $\Psi \rightarrow$ N–C$_\alpha$–C(O)–N and $\Phi \rightarrow$ C(O)–N–C$_\alpha$–C(O) dihedral angles (Figure S1) from 0° to 360° in steps of 10° at the B3LYP/6-31+G(d) level of theory in *vacuo* and in combination with the Polarizable Continuum Model (PCM), using the Gaussian 16 program.(2)

The conformational preferences of these two blocked amino acids have been extensively studied in the gas phase, and in solution, most notably the C7 and C5 conformers, arising from the intermolecular hydrogen bonds (HBs) that lead to the formation of a seven or five-membered rings.(3, 4, 5, 6, 7, 8, 9) The reported in literature β_2, *C5* and *C7* were identified as the lowest energy minima. We also consider dimeric forms for NAGMA and NALMA. Optimizations and frequency calculations were carried out at the B3LYP/6-311++G(d,p) level for all conformers (and dimers) and for the structure designed by saturating the potential hydrogen bond (HB) sites in the dipeptides. In what follows, that motif will be called "*Supermolecule + 4W*". Orbital interactions associated to hydrogen bonding were analyzed in the Natural Bond Orbitals (NBO) framework(10, 11) and the interaction energies were obtained via second order perturbation corrections to the Fock matrix with the NBO7 program.(12)

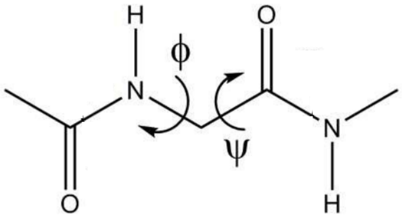

Figure S1: Dipeptide model dihedral angle representations

2.2 MD simulations

Starting from the β_2 conformer, 30 ns long Molecular Dynamics simulations of dipeptides in solution were performed with the GROMACS code(13). Geometrical and Lennard-Jones parameters for NAGMA and NALMA were obtained from the General Amber Force Field(14) and partial atomic charges were derived with the Charge Model 5(15). Virtual sites were added to the carbonyl groups in order to improve the directionality of the hydrogen bonds to be formed with the solvent molecules. The squared simulation boxes were filled with TIP3P water molecules.(16) After energy minimization and NVT and NPT equilibrations, the MD simulation was performed at 300 K and 1 atm with periodic boundary conditions, particle-mesh Ewald summation(17) for long-range electrostatics, 12 Å cutoff for nonbonded inter-

actions, constrained X–H bonds, and 2 fs time step. A modified Berendsen thermostat(18) and a Parinello-Rahman barostat(19), with a coupling constant, τ, of 0.1 ps for each, were employed to maintain temperature and pressure. Atomic coordinates were saved every 10 ps. We skipped the first 10 ns of the trajectory, and extracted about 200 snapshots selecting the closest water molecules within a radius of 8 Å of the peptide to obtain solute concentrations of ≈20 mg/mL (corresponding to a molar ratio of 1:336 peptide:H_2O)) similar to those in the experiments. Hydration patterns were analyzed with the TRAVIS package(20, 21). The clustering method reported in Ref.22 was used to identify similar conformations sampled during the MD run. Cutoffs were set to be the averages of the RMSD.

2.3 Calculations of the Spectra

We performed optimizations on geometries coming from the MD trajectories, and then carried out TD-DFT, frequencies and Raman calculations. In those calculations, solvent effects were described by means of the Quantum Mechanics/Fluctuating Charges (QM/FQ) model(23), applied to each of the extracted snapshots. Different FQ parameterizations(24, 25) were exploited.

For the Resonance Raman spectra, we used the Franck Condon Vertical Gradient (FC|VG) approach in which the vibrational frequencies and normal modes of the excited state are assumed to be the same as the ground state, and the transition dipole moments are considered to be independent of the molecular geometry. Also, only the energy gradients are computed for the excited state.(26) We calculated the rotational invariants of the polarizability tensor using the most common set that consists of the mean polarizability a, the antisymmetric anisotropy δ and the anisotropy g. For symmetric tensors, $\delta = 0$. a and g have the following general definitions when using the summation convention:

$$a_i^2 = \frac{1}{9}\sum_{ab} \alpha_{aa}^{i*}\alpha_{bb}^{i} = \frac{1}{9}|\alpha_{xx}^{i} + \alpha_{yy}^{i} + \alpha_{zz}^{i}|^2 \tag{S1}$$

$$g_i^2 = \frac{1}{2}\sum_{ab}(3\alpha_{ab}^{i*}\alpha_{ab}^{i} - \alpha_{aa}^{i*}\alpha_{bb}^{i}) \tag{S2}$$

where α_{ab} denotes the components of the complex Raman polarizability tensor. The above invariants are related to the scattered intensity by means of equations defining the cross section for given experimental setups. For the commonly employed 90° scattering geometry, the Raman differential cross section, σ_i, is written as follows(27, 28)

$$\sigma_i = \left(\frac{\omega - \omega_i}{c}\right)^4 \frac{45a_i^2 + 7g_i^2}{45} \tag{S3}$$

where ω is the frequency of the incident light. In such a geometry, the incident linearly

polarized radiation is perpendicular to the scattering plane and a detection of all scattered polarizations is located at an angle of 90° with respect to the incident radiation direction.

Reported averaged absorption, Raman and RR spectra spectra were obtained by convoluting peak intensities with Gaussian or Lorentzian functions. For the Gaussian functions we used a full width at half maximum (FWHM) of 0.5 eV, whereas for the Lorentzian ones we chose an FWHM of 20 cm^{-1}. RR Excitation profiles were also computed by scanning different excitations wavelengths based on the information provided by the UV-Vis absorption spectra. All QM/FQ spectra calculations were conducted using a locally modified version of the Gaussian 16 package at the B3LYP/6–311++G(d, p) level. In the case of QM/FQ RR spectra, some extra computations were carried out with CAM-B3LYP, PBE0, and M06-2X functionals, and it turned out that B3LYP fits better for the particular systems studied in this work.

Electronic absorption, Raman and Resonance Raman spectra were also calculated on the conformers found through the 2D scans of the Ramachandran angles of the peptides, using geometries both in the gas phase and in PCM.

3 Structural analysis

Table S1: NAGMA and NALMA molecular structures for all the conformations and environments in the study.

**NAGMA**	**NALMA**
**Gas phase, *C5***<br>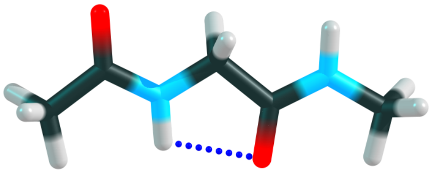	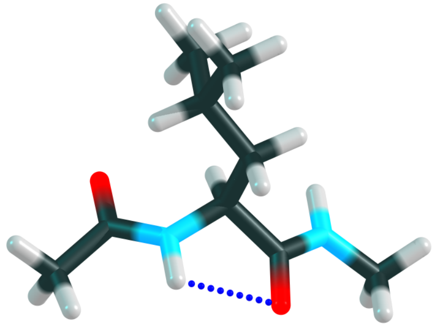
**Gas phase, *C7***<br>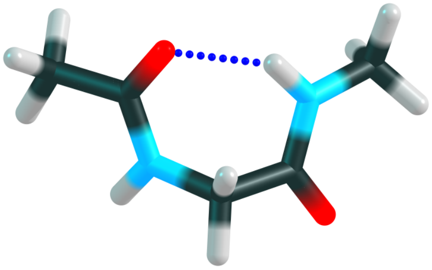	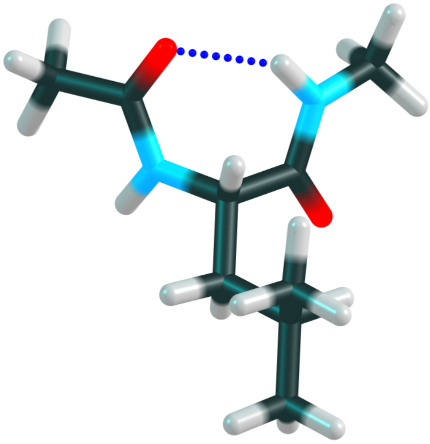
**Gas phase,** β_2<br>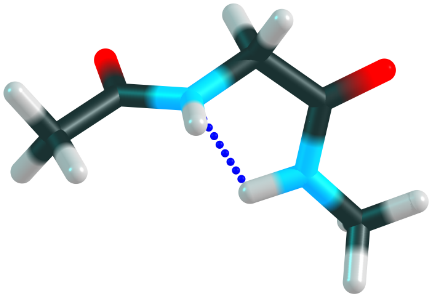	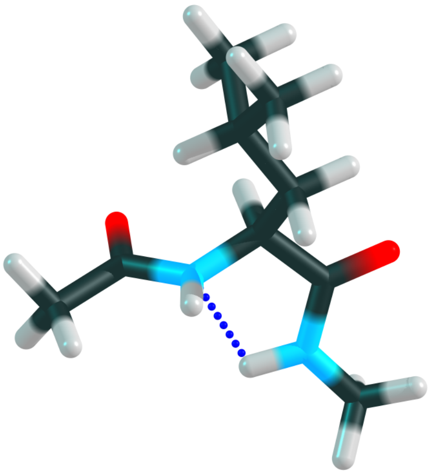

Table S1: NAGMA and NALMA molecular structures for all the conformations and environments in the study.

NAGMA	NALMA
**PCM, *C5***<br>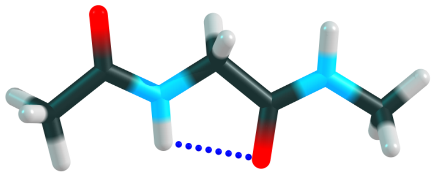	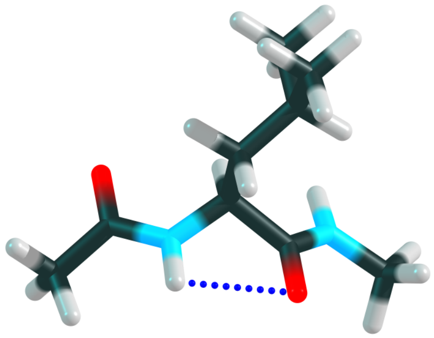
**PCM, *C7***<br>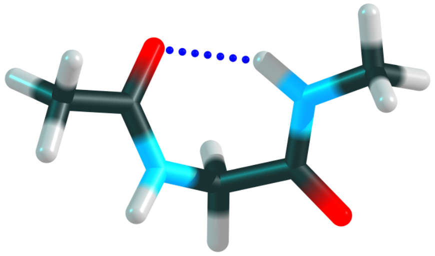	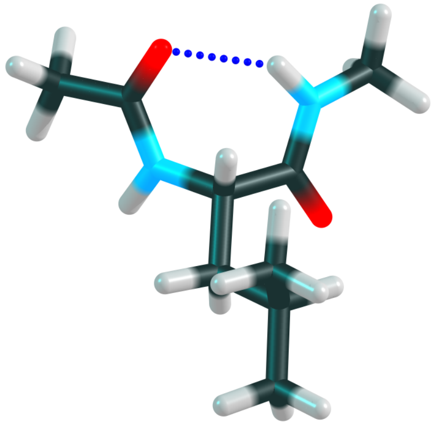
**PCM,** β_2<br>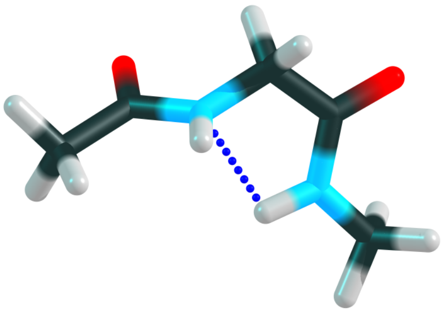	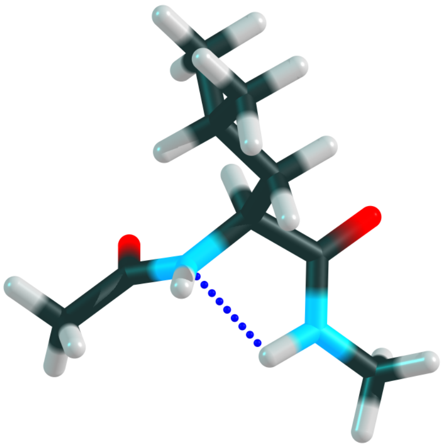

Table S1: NAGMA and NALMA molecular structures for all the conformations and environments in the study.

NAGMA	NALMA
**Supermolecule + 4W PCM**	
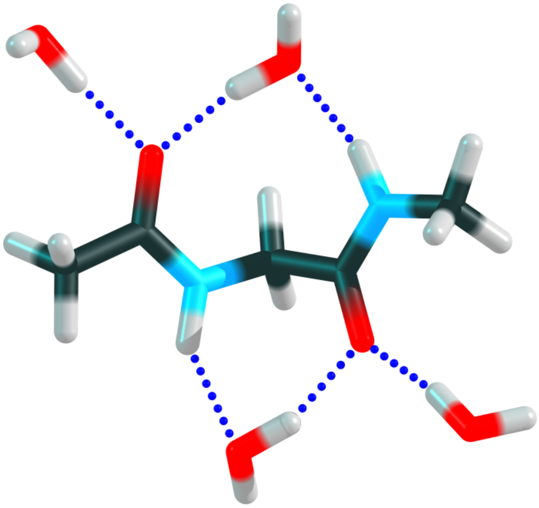	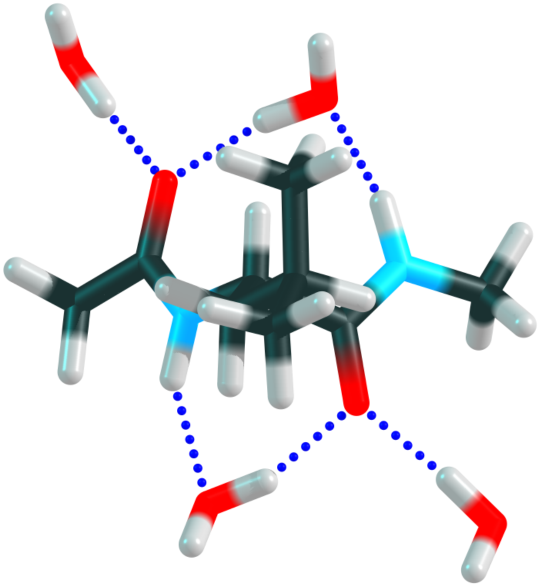
**QM/FQ**	
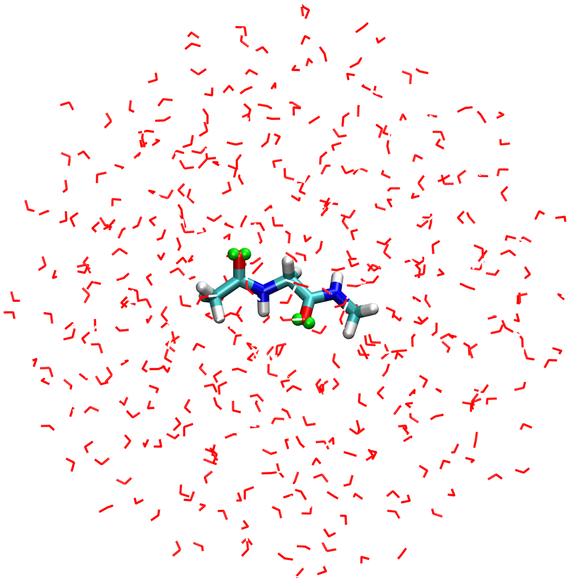	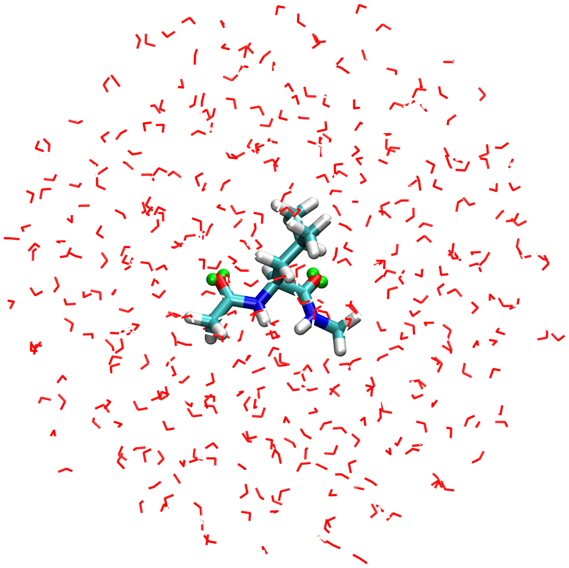

Table S1: NAGMA and NALMA molecular structures for all the conformations and environments in the study.

<table>
<tr><th>NAGMA</th><th>NALMA</th></tr>
<tr><td>Dimer
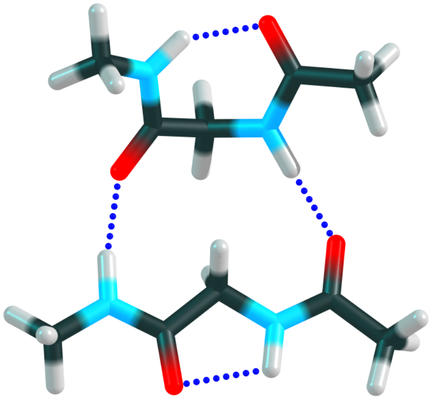</td><td>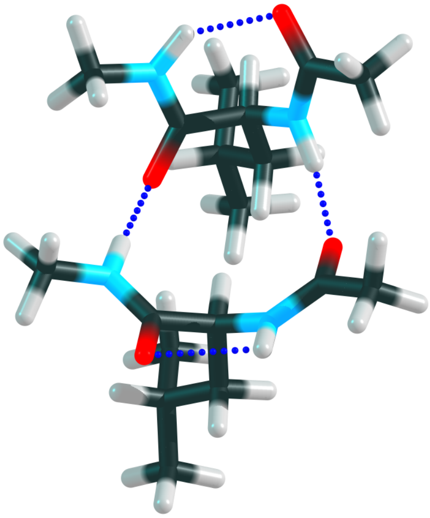</td></tr>
<tr><td>Dimer solvated
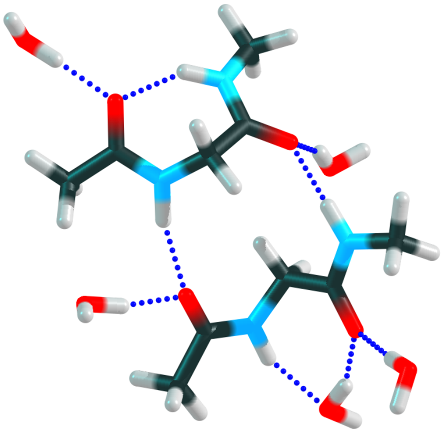</td><td>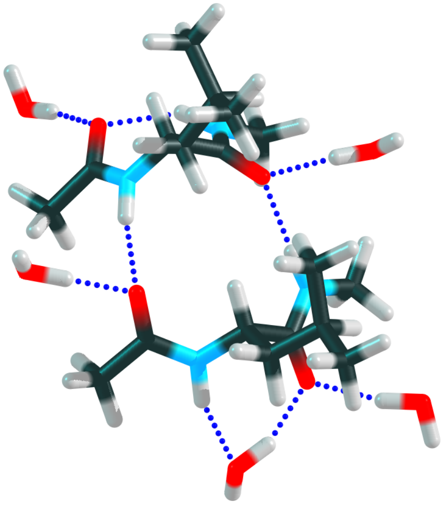</td></tr>
</table>

Table S2: Structural parameters for NAGMA at the B3LYP/6-311++G(d, p) level of theory. Distances are reported in Å and dihedral angles in degrees. In the MD case average distances are listed.

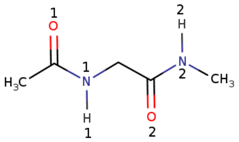

Motif	Φ	Ψ	C=O1	C–N1	N1–H1	C=O2	C–N2	N2–H2	O1···H2	O2···H1	N1···H2	N2···H1
Gas phase, *C5*	-180.0	-180.0	1.224	1.360	1.011	1.223	1.358	1.008		2.186		
Gas phase, *C7*	-82.6	70.8	1.228	1.361	1.007	1.223	1.355	1.015	2.083			
Gas phase, β_2	-113.3	16.7	1.218	1.377	1.008	1.221	1.358	1.008			2.365	
PCM, *C5*	180.0	-180.0	1.235	1.350	1.012	1.233	1.344	1.009		2.185		
PCM, *C7*	83.1	-61.6	1.237	1.353	1.009	1.233	1.345	1.015	2.050			
PCM, β_2	-99.9	0.00	1.230	1.365	1.009	1.233	1.343	1.009			2.340	
PCM, 4W	103.8	-115.6	1.248	1.343	1.018	1.248	1.331	1.022				
MD			1.218	1.379	1.012	1.218	1.379	1.012	4.234	3.734	2.510	2.772
Dimer	-81.6	70.3	1.236	1.347	1.014	1.228	1.345	1.018		2.162		
	-178.2	179.3	1.235	1.355	1.021	1.235	1.345	1.019	1.982			
Dimer solvated	97.8	-147.9	1.245	1.344	1.018	1.244	1.335	1.020				
	-81.8	68.6	1.243	1.352	1.017	1.244	1.336	1.019	1.968			

Table S3: Structural parameters for NALMA at the B3LYP/6-311++G(d, p) level of theory. Distances are reported in Å and dihedral angles in degrees. In the MD case average distances are listed.

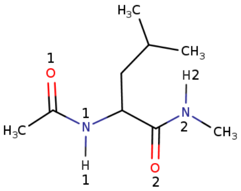

Motif	Φ	Ψ	C=O1	C–N1	N1–H1	C=O2	C–N2	N2–H2	O1···H2	O2···H1	N1···H2	N2···H1
Gas phase, *C5*	-128.8	150.1	1.224	1.365	1.011	1.224	1.358	1.008		2.307		
Gas phase, *C7*	-83.7	79.7	1.229	1.361	1.009	1.223	1.356	1.015	2.127			
Gas phase, β_2	-112.3	9.5	1.218	1.376	1.010	1.222	1.358	1.008			2.283	
PCM, *C5*	-140.7	130.2	1.234	1.355	1.010	1.234	1.345	1.008		2.479		
PCM, *C7*	-85.8	79.6	1.237	1.354	1.010	1.231	1.349	1.014	2.175			
PCM, β_2	-93.8	-9.5	1.230	1.363	1.010	1.234	1.344	1.008			2.298	
PCM, 4W	-108.7	111.6	1.249	1.343	1.019	1.248	1.332	1.021				
MD			1.218	1.379	1.012	1.218	1.379	1.012	3.952	3.998	2.358	2.881
Dimer	-133.0	131.9	1.234	1.350	1.011	1.229	1.348	1.018		2.434		
	-84.1	81.4	1.234	1.357	1.019	1.234	1.349	1.017	2.085			
Dimer solvated	-134.3	122.5	1.245	1.343	1.020	1.246	1.333	1.017				
	-85.4	78.2	1.243	1.352	1.018	1.243	1.340	1.017	2.067			

3.1 Ramachandran plots

Figure S2 displays the bidimensional scan for the Φ and Ψ torsion angles of the dipeptides backbone and the dihedral distribution functions and time evolution during the MD

sampling. From these plots it is evident that there are many possibilities for the angle combinations that have low energies (regions in blue in the 2D scans in Figure S2) thus stabilizing the dipeptides, while other Φ/Ψ couples are not possible for steric hindrance. In fact, the leucine residue causes the conformational space for NALMA to be more restricted than for NAGMA, with higher penalties of up to 22 kcal/mol in the forbidden (inaccessible) regions. Correspondingly, our MD runs suggest that for NAGMA the Ψ sampled values range from -60 to 60 degrees and the most populated Φ angles are around ±180 degrees, while for NALMA these torsion angles are limited to the (-60°-30°) interval for Ψ, and the most occurring Φ value is around -120°.

3.2 Hydration patterns

3.2.1 RDFs

Since most conformers show a C=O$\cdots$H intramolecular distance of about 3.9 Å (see top panel in Figure S3), the competitive effect seems to be dominated by the interaction with the solvent molecules, from which C=O$\cdots$H$_w$ and N–H$\cdots$O$_w$ HBs arise. As a result, in the first solvation shell, 2 and 1 water molecules for each C=O$\cdots$H$_w$, N–H$\cdots$O$_w$ contact are found to be coordinated to the dipeptides. In contrast, the nitrogen atom of the dipeptides does not interact with the hydrogen atoms of the surrounding water molecules.

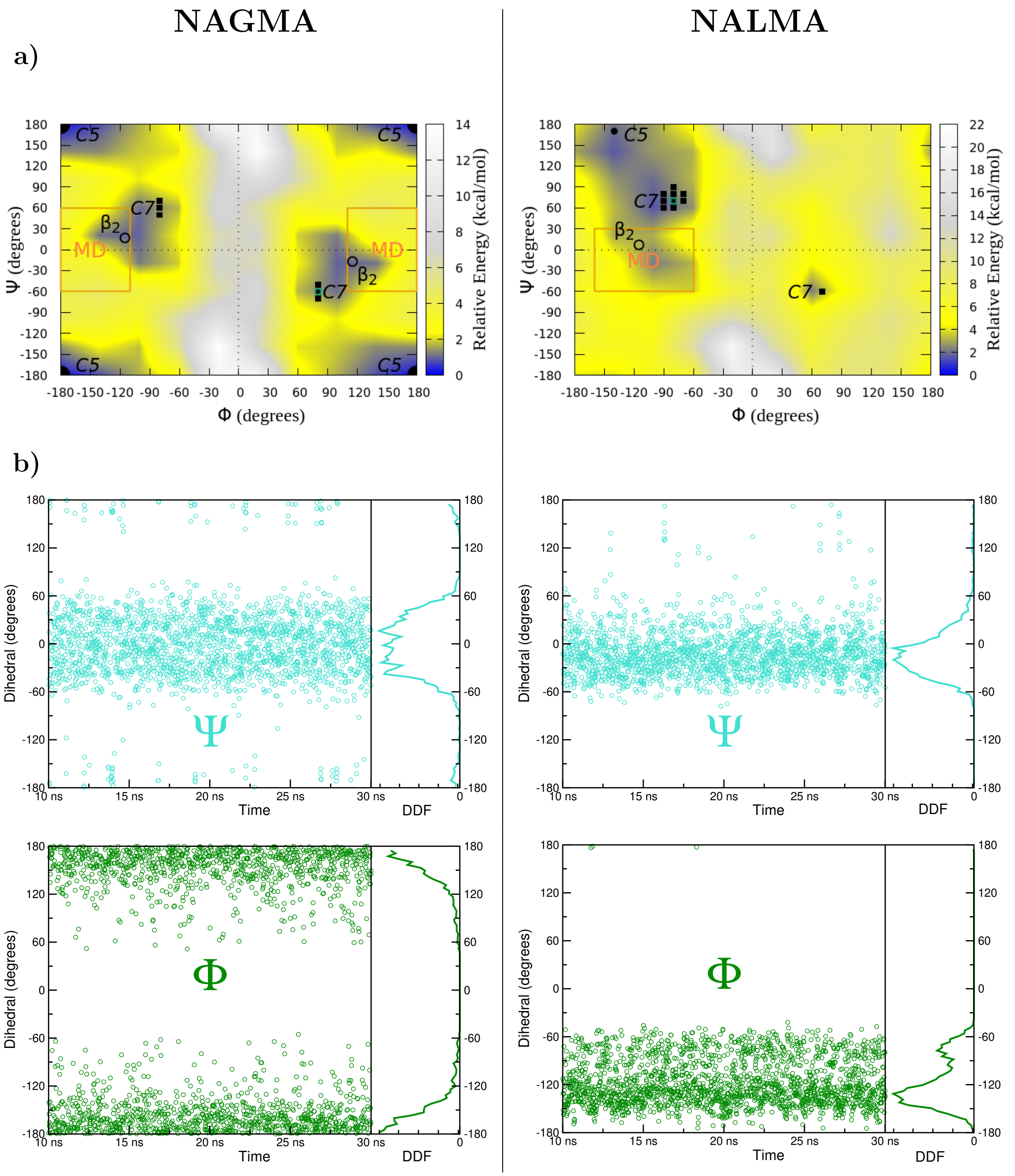

Figure S2: **a)** 2D scans of the torsion angles shown in Figure S1, calculated using the model chemistry B3LYP/6-31G(d)/PCM. **b)** Dihedral Distribution Functions (DDFs) and time development of the Ramachandran angles in the 10–30 ns MD interval. The torsion angles for the *C5*, *C7* and β_2 monomers used in this work are are highlighted in thick black points in every plot.

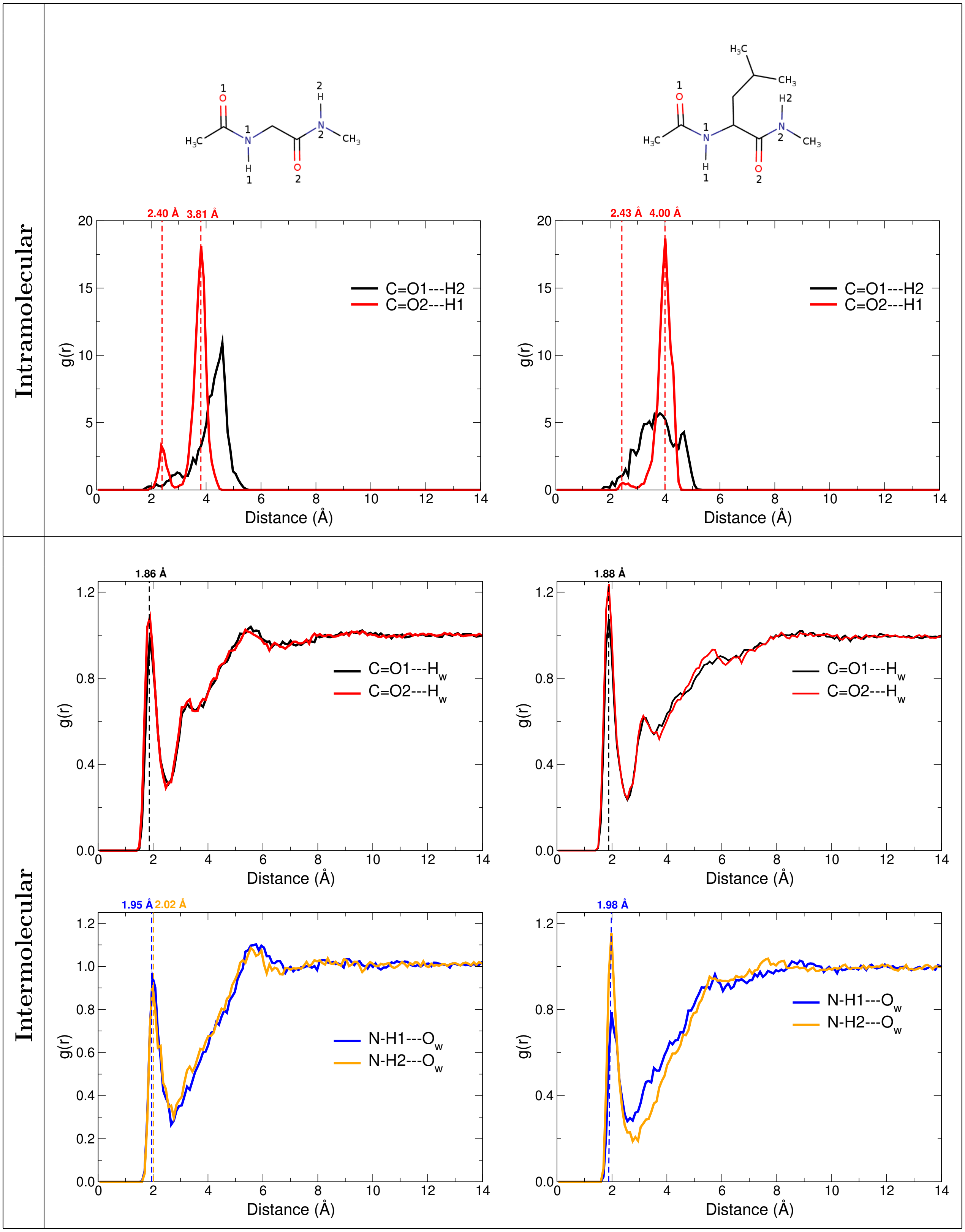

Figure S3: Radial Distribution Functions (RDFs) for the Intramolecular and intermolecular O$\cdots$H interactions in solvated dipeptides, NAGMA (left) and NALMA (right).

3.2.2 NBO interactions

Stabilization energies are depicted in Figure S4 with the corresponding motifs. It is clear that $E^{(2)}_{\rm d-a}$ values for intramolecular HBs are smaller when compared to those in the intermolecular cases, either with water molecules or with another peptide monomer. This could explain the fact that the β_2 conformer was found to be the lowest energy structure in solution, since it leaves the carbonyl groups available to interact with the solvent, maximizing the orbital interactions. In the supermolecule cases, notice that $\sum E^{(2)}_{\rm d-a,NAGMA} > \sum E^{(2)}_{\rm d-a,NALMA}$ indicating that NAGMA interacts more strongly than NALMA with water molecules.

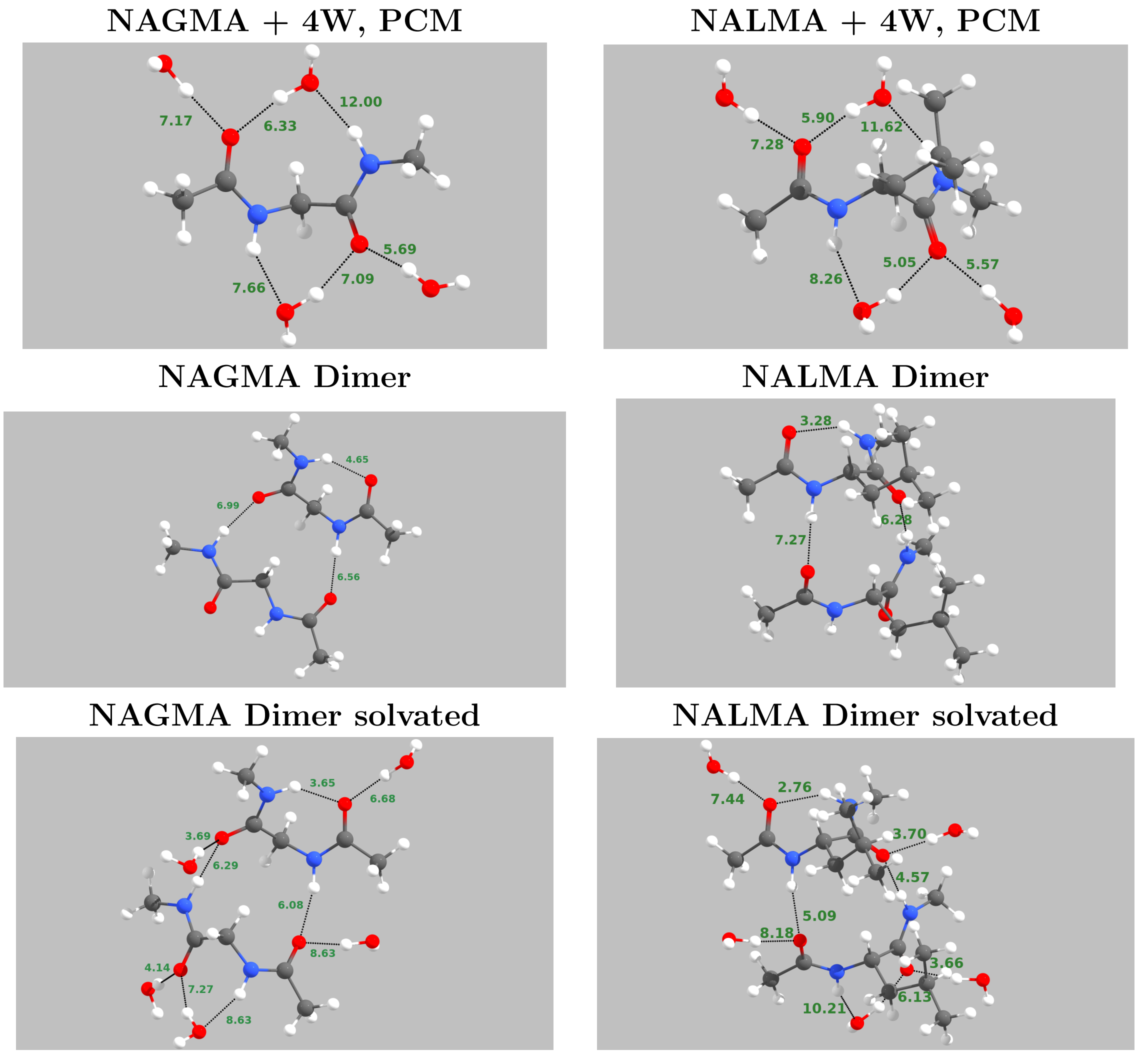

Figure S4: Intermolecular orbital interaction energies (in kcal/mol) for microsolvated and dimeric forms of NAGMA and NALMA. All interactions are of the type $n_O \rightarrow \sigma_{\rm O-H}$ or $n_O \rightarrow \sigma_{\rm N-H}$. Orbitals are omitted for visualization purposes.

4 Spectra and further analysis

4.1 Computed UV/Vis absorption spectra

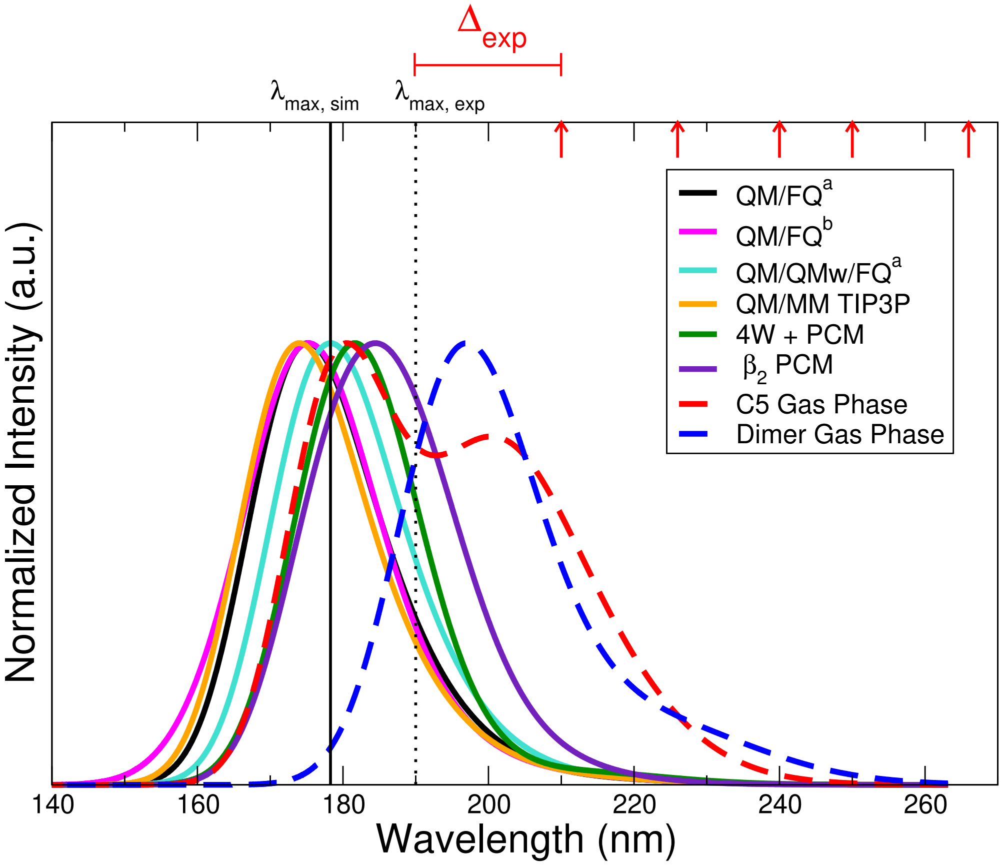

Figure S5: Computed UV-Vis absorption spectrum for NAGMA in different environments. Vertical lines mark the position of the absorption maxima for the simulated, $\lambda_{max,sim}$ at 175 nm, and for the experimental spectra, $\lambda_{max,exp}$ at 190 nm. Red arrows indicate the wavelengths at which RR spectra were measured and Δ_{exp} is the difference between $\lambda_{max,exp}$ and one the experimental wavelengths used in RR. This Δ_{exp} is to be taken into account in the RR calculations. [a]FQ parametrization from Ref. 24, [b]FQ parametrization from Ref. 25.

Orbitals involved in the main transitions

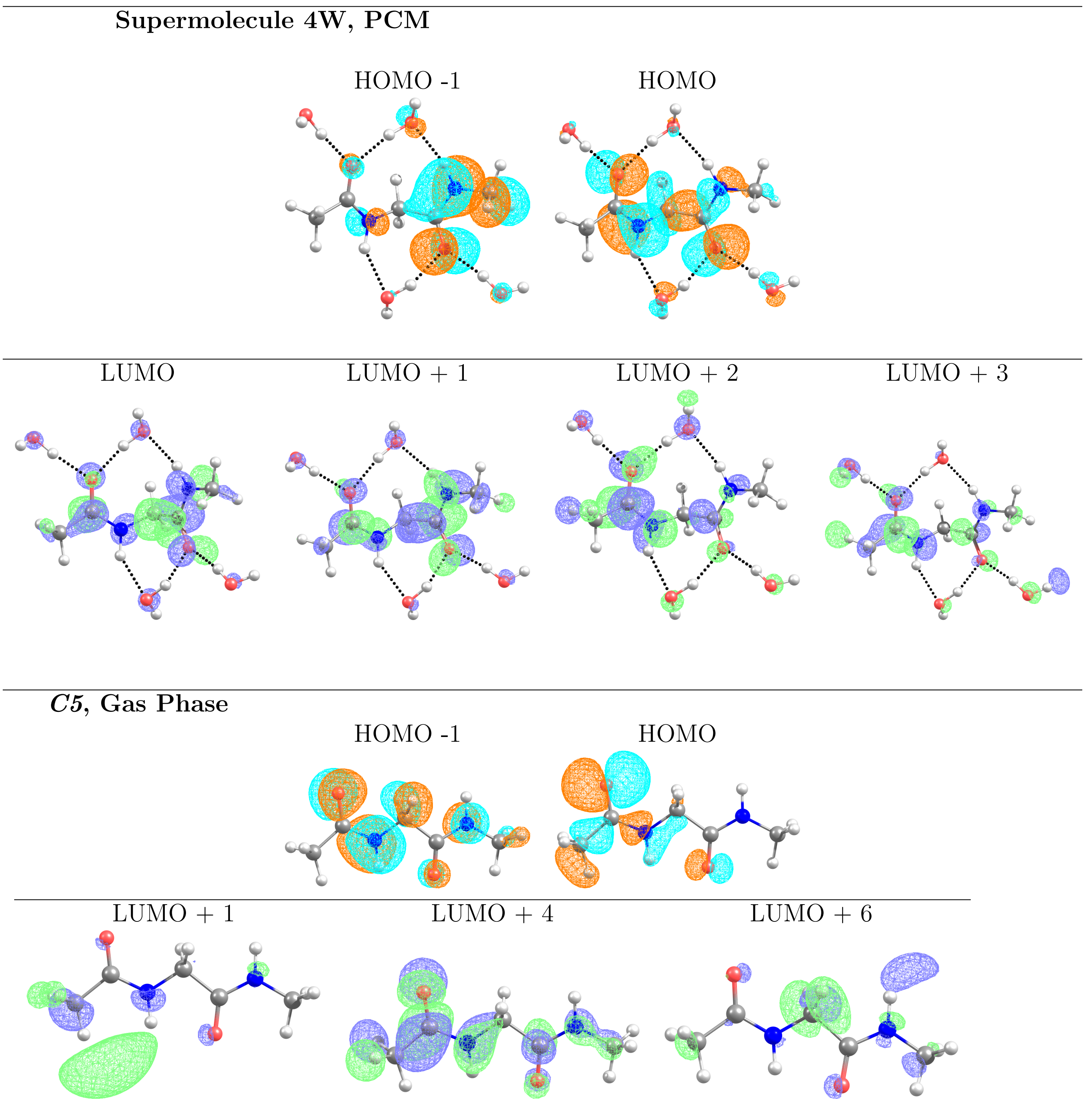

Figure S6: Orbitals of NAGMA involved in the $\pi \rightarrow \pi^*$ transitions.

4.2 Normal modes

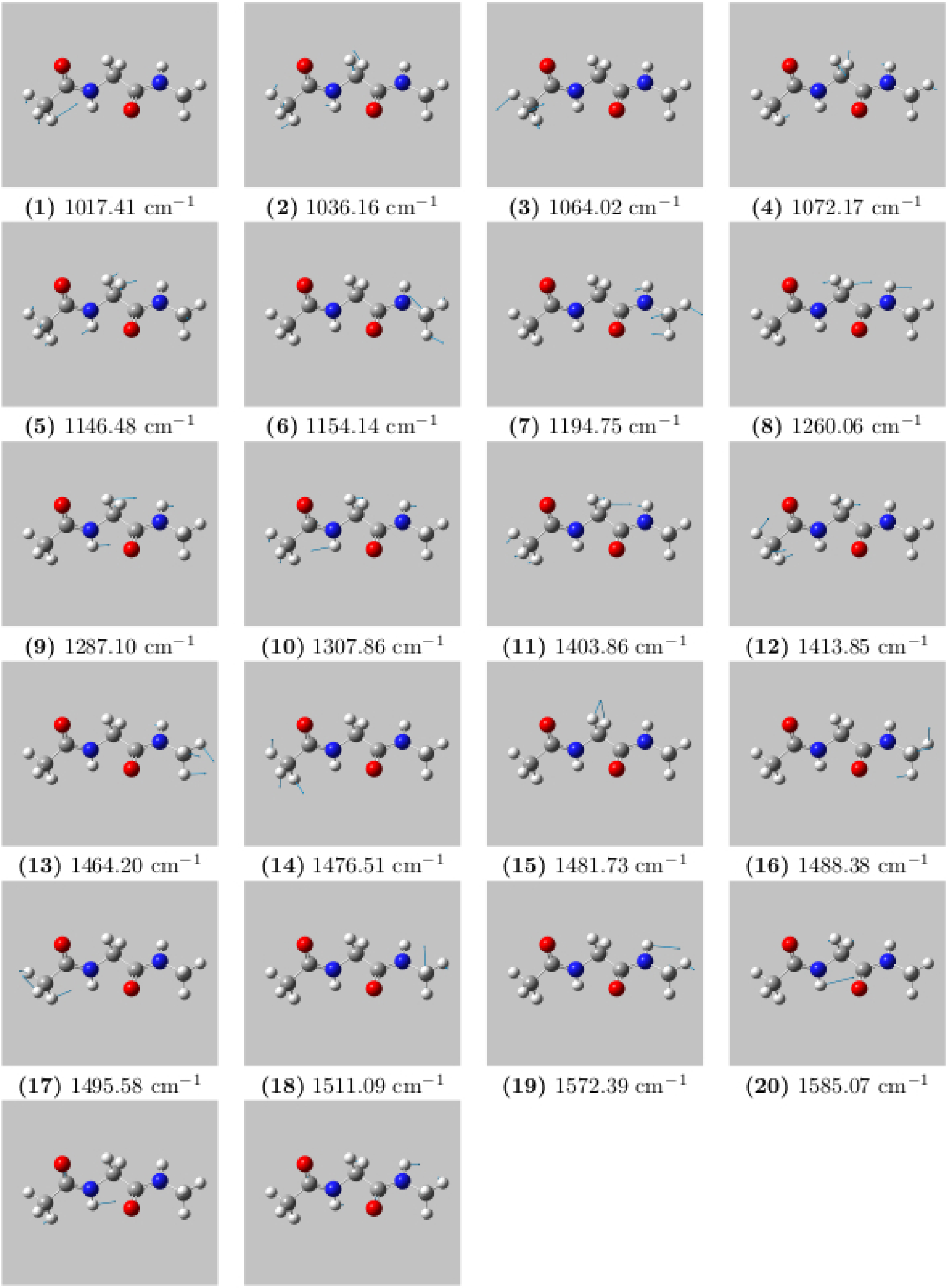

(1) 1017.41 cm^{-1} (2) 1036.16 cm^{-1} (3) 1064.02 cm^{-1} (4) 1072.17 cm^{-1}

(5) 1146.48 cm^{-1} (6) 1154.14 cm^{-1} (7) 1194.75 cm^{-1} (8) 1260.06 cm^{-1}

(9) 1287.10 cm^{-1} (10) 1307.86 cm^{-1} (11) 1403.86 cm^{-1} (12) 1413.85 cm^{-1}

(13) 1464.20 cm^{-1} (14) 1476.51 cm^{-1} (15) 1481.73 cm^{-1} (16) 1488.38 cm^{-1}

(17) 1495.58 cm^{-1} (18) 1511.09 cm^{-1} (19) 1572.39 cm^{-1} (20) 1585.07 cm^{-1}

(21) 1696.85 cm^{-1} (22) 1717.57 cm^{-1}

Figure S7: Normal modes of solvated NAGMA (using QM/FQ model), with their corresponding frequencies in the 1000–1800 cm^{-1} interval. These modes are referred to one random snapshot (188)

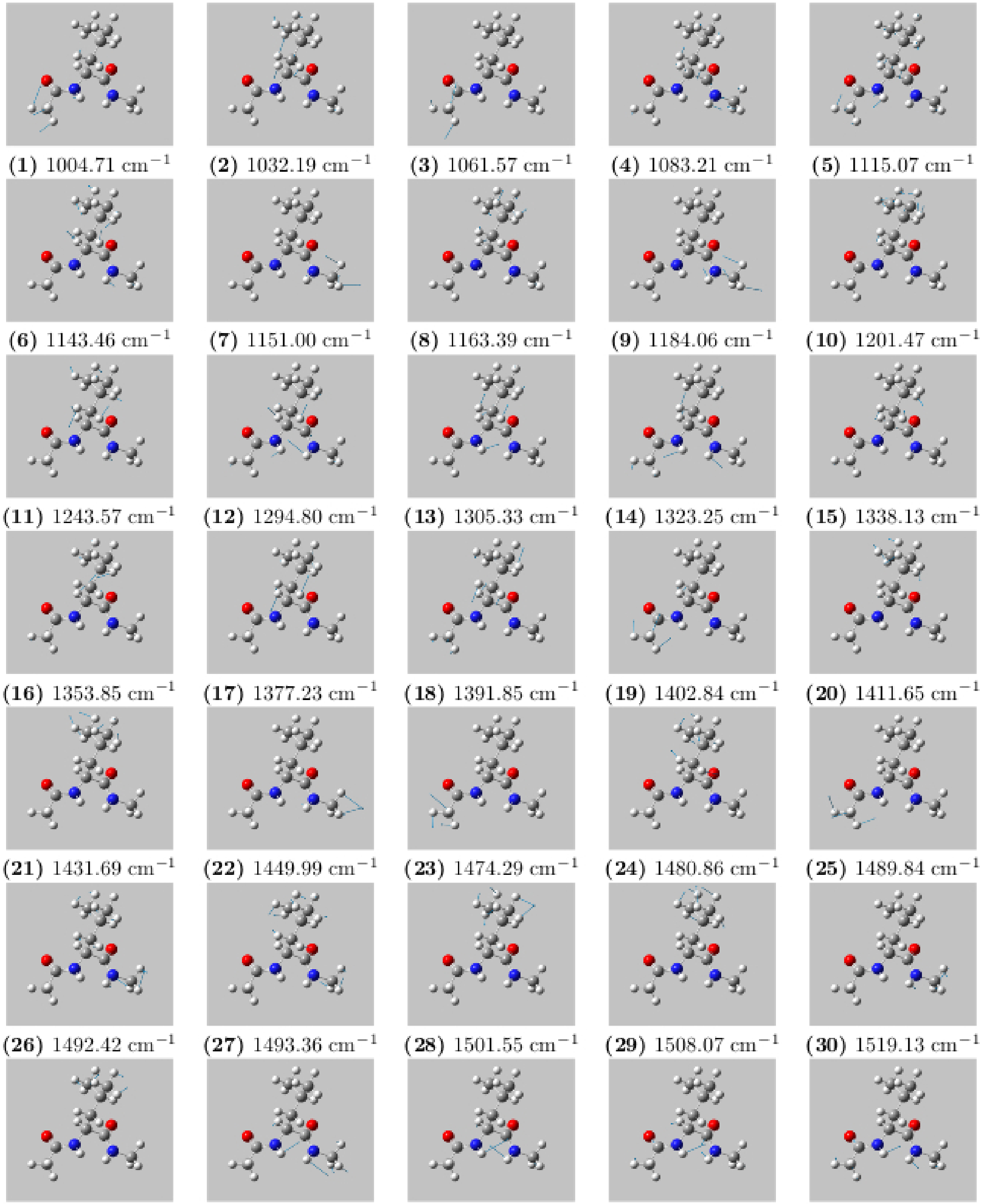

(**1**) 1004.71 cm^{-1} (**2**) 1032.19 cm^{-1} (**3**) 1061.57 cm^{-1} (**4**) 1083.21 cm^{-1} (**5**) 1115.07 cm^{-1}
(**6**) 1143.46 cm^{-1} (**7**) 1151.00 cm^{-1} (**8**) 1163.39 cm^{-1} (**9**) 1184.06 cm^{-1} (**10**) 1201.47 cm^{-1}
(**11**) 1243.57 cm^{-1} (**12**) 1294.80 cm^{-1} (**13**) 1305.33 cm^{-1} (**14**) 1323.25 cm^{-1} (**15**) 1338.13 cm^{-1}
(**16**) 1353.85 cm^{-1} (**17**) 1377.23 cm^{-1} (**18**) 1391.85 cm^{-1} (**19**) 1402.84 cm^{-1} (**20**) 1411.65 cm^{-1}
(**21**) 1431.69 cm^{-1} (**22**) 1449.99 cm^{-1} (**23**) 1474.29 cm^{-1} (**24**) 1480.86 cm^{-1} (**25**) 1489.84 cm^{-1}
(**26**) 1492.42 cm^{-1} (**27**) 1493.36 cm^{-1} (**28**) 1501.55 cm^{-1} (**29**) 1508.07 cm^{-1} (**30**) 1519.13 cm^{-1}
(**31**) 1522.90 cm^{-1} (**32**) 1565.51 cm^{-1} (**33**) 1605.71 cm^{-1} (**34**) 1712.06 cm^{-1} (**35**) 1714.81 cm^{-1}

Figure S8: Normal modes of solvated NALMA (using QM/FQ model), with their corresponding frequencies in the 1000–1800 cm^{-1} interval. These modes are referred to one random snapshot (188)

4.3 Simulated UVRR spectra for NAGMA and NALMA in aqueous solution

Choice of the model chemistry for the RR calculations: A comparison of the performance of different DFT functionals in the calculation of the QM/FQ RR spectra of the most representative conformer sampled during the MD of aqueous NAGMA is shown here below.

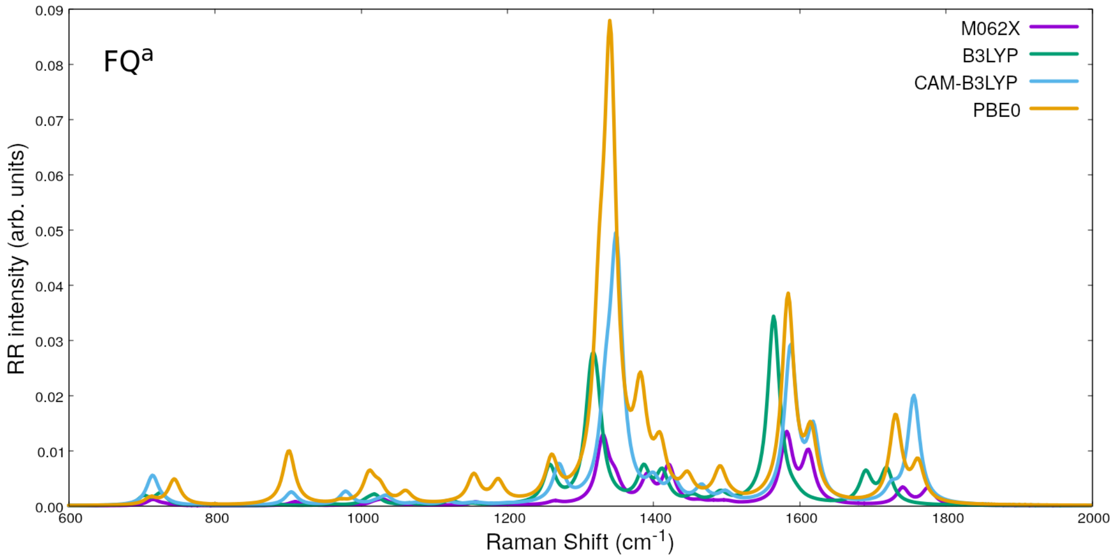

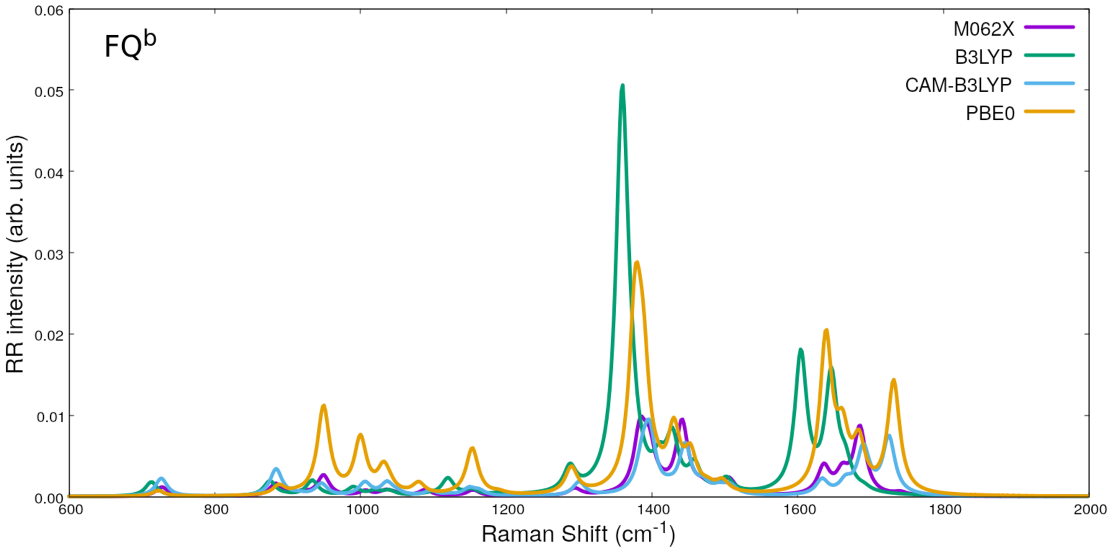

Overall, there are large differences when comparing the spectra to those obtained using B3LYP. With this latter, in both FQ parametrizations, spectra resemble more the experimental data that we collected. Therefore, we chose B3LYP functional for the entire set of calculations presented in the paper.

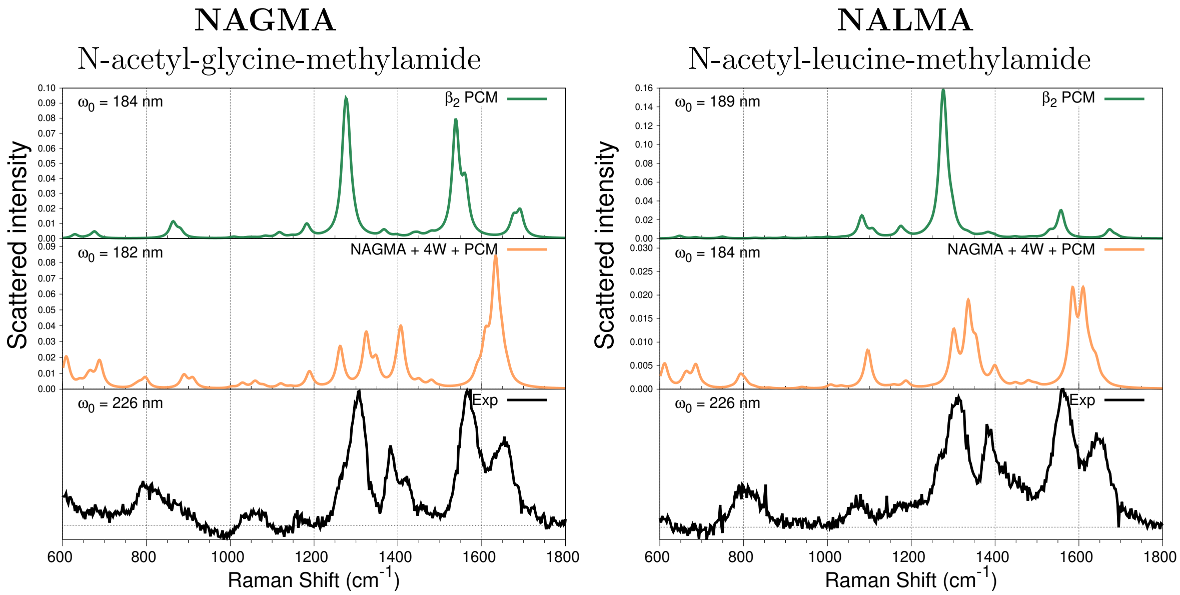

Figure S9: UV-resonance Raman spectra of NAGMA (left) and NALMA (right) in different representations of the aqueous solution, calculated when their corresponding absorption maxima wavelengths are used to irradiate the system (see Table 1). β_2 is the lowest energy conformer of NAGMA and NALMA in solution. RR intensities (in $cm^2mol^{-1}sr^{-1}$) were calculated with a damping factor of 200 cm^{-1} and broadened using Lorentzian functions with FWHM = 20 cm^{-1}. Experimental spectra were collected using 226 nm as excitation wavelength on aqueous solutions of dipeptides (1:366 peptide:H_2O).

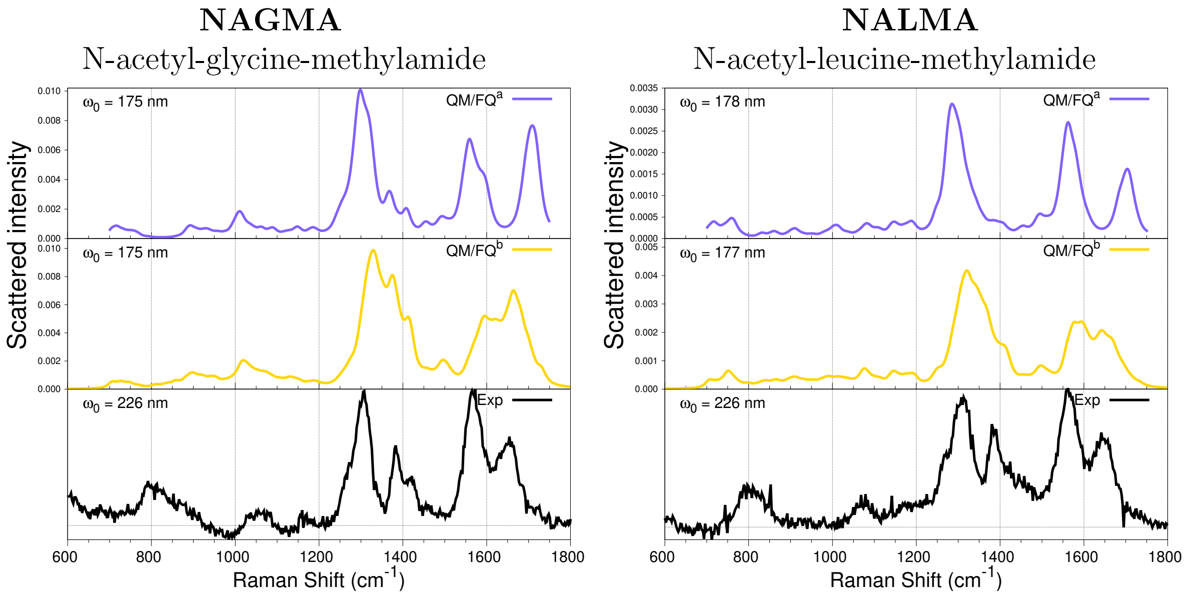

Figure S10: QM/FQ UV-resonance Raman spectra of NAGMA (left) and NALMA (right) using different parametrizations and calculated when their corresponding absorption maxima wavelengths are used to irradiate the system (see Table 1). [a]FQ parametrization from Ref. 24, [b]FQ parametrization from Ref. 25. RR intensities (in $cm^2 mol^{-1} sr^{-1}$) were calculated with a damping factor of 200 cm^{-1} and broadened using Lorentzian functions with FWHM = 20 cm^{-1}. Experimental spectra were collected using 226 nm as excitation wavelength on aqueous solutions of dipeptides (1:366 peptide:H_2O).

4.4 Experimental UVRR spectra of the peptides in their microcrystalline form

(a)

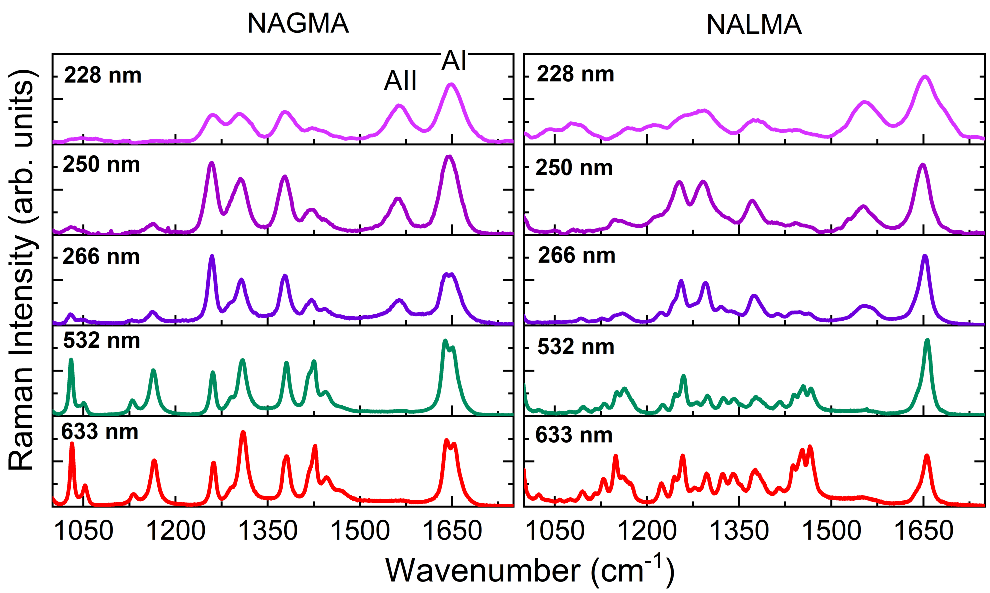

(b)

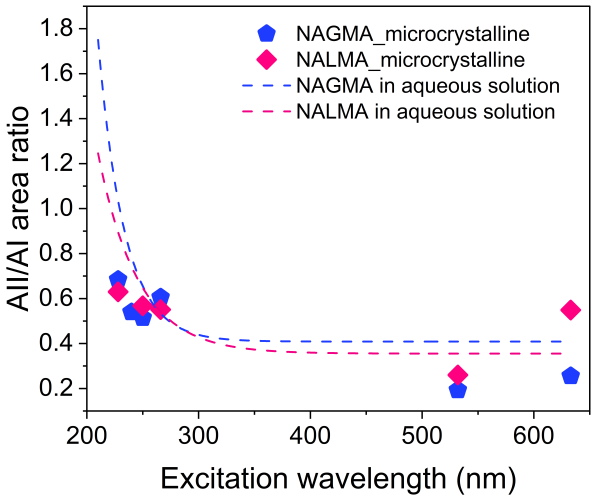

Figure S11: Experimental results of NAGMA and NALMA in their microcrystalline form (a) Raman spectra collected using different excitation wavelengths ranging from visible to deep UV energies. (b) estimated ratio of the areas of amide modes AII/AI as a function of the excitation wavelength.

4.5 Natural Resonance Theory (NRT) Analysis

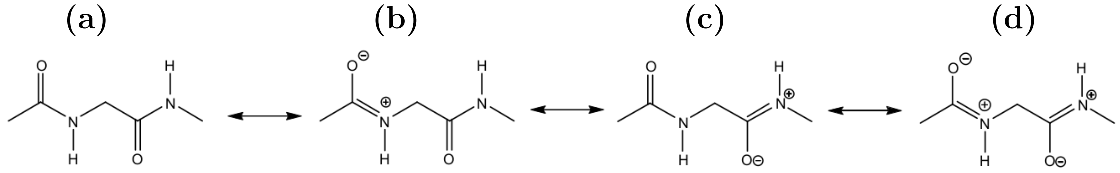

Figure S12: Main resonance contributors to the NAGMA structure obtained from the NRT analysis

Table S4: Percentage values of NAGMA main resonance structures shown in Figure S12, obtained from the NRT analysis of the different conformers

	**(a)**	**(b)**	**(c)**	**(d)**
Gas phase, *planar, C5*	22.94	17.97	18.93	13.96
Gas phase, *bent, C7*	17.17	15.99	15.39	14.19
Gas phase, β_2	23.12	17.06	19.10	13.10
PCM, *planar, C5*	21.30	18.70	19.48	16.88
PCM, *bent, C7*	18.57	18.58	16.31	16.33
PCM, β_2	21.30	18.36	19.79	16.85
Cluster 4W + PCM	16.53	19.38	18.09	21.02

4.6 Simulated RR Excitation Profiles

All spectra are scaled such that the maximum intensity is unity. A Lorentzian broadening with a FWHM of 20 cm^{-1} was used.

4.6.1 NAGMA

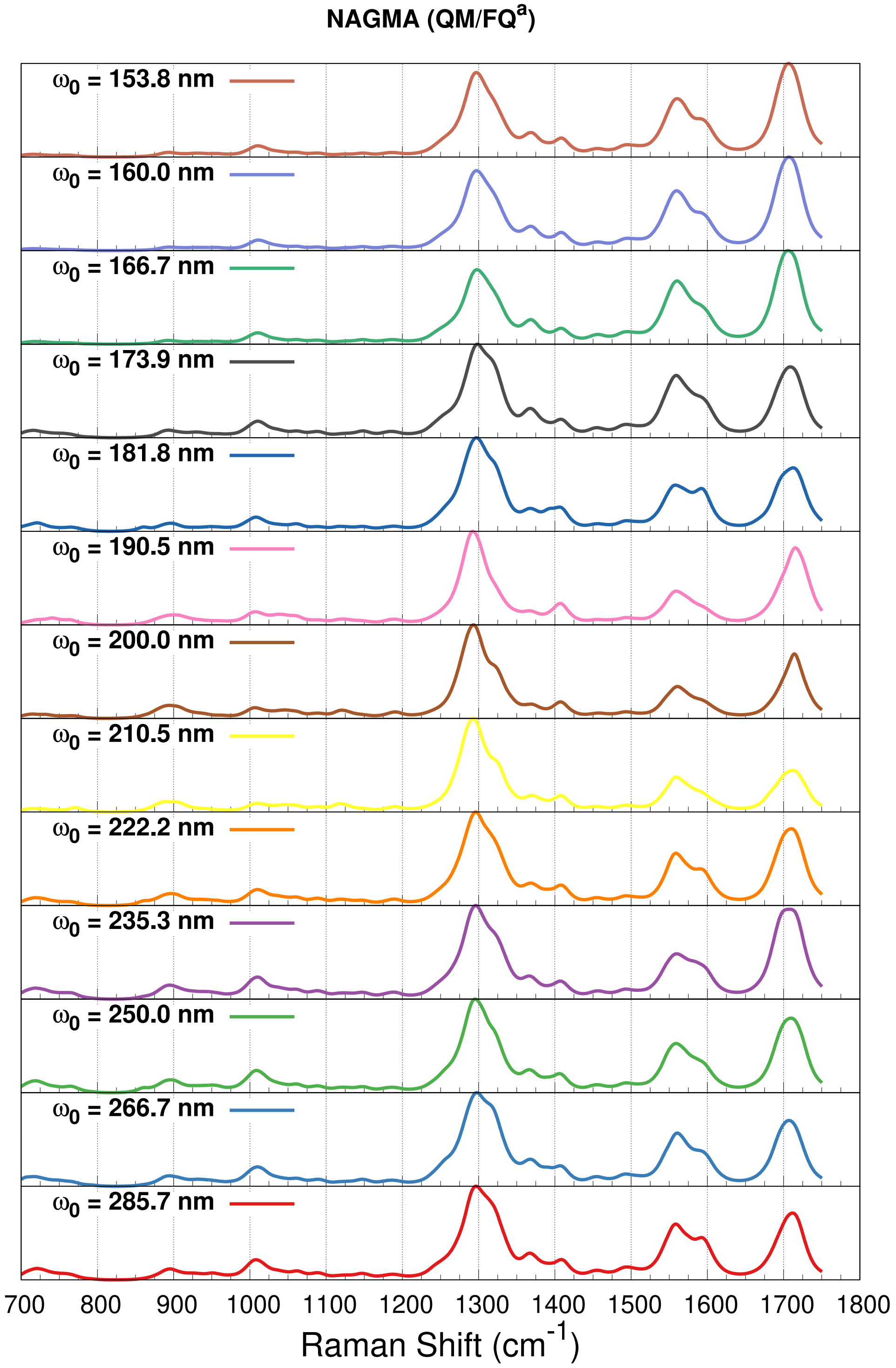

Figure S13: QM/FQ Resonance Raman Excitation profile of NAGMA in aqueous solution. FQ parameters were taken from Ref. 24.

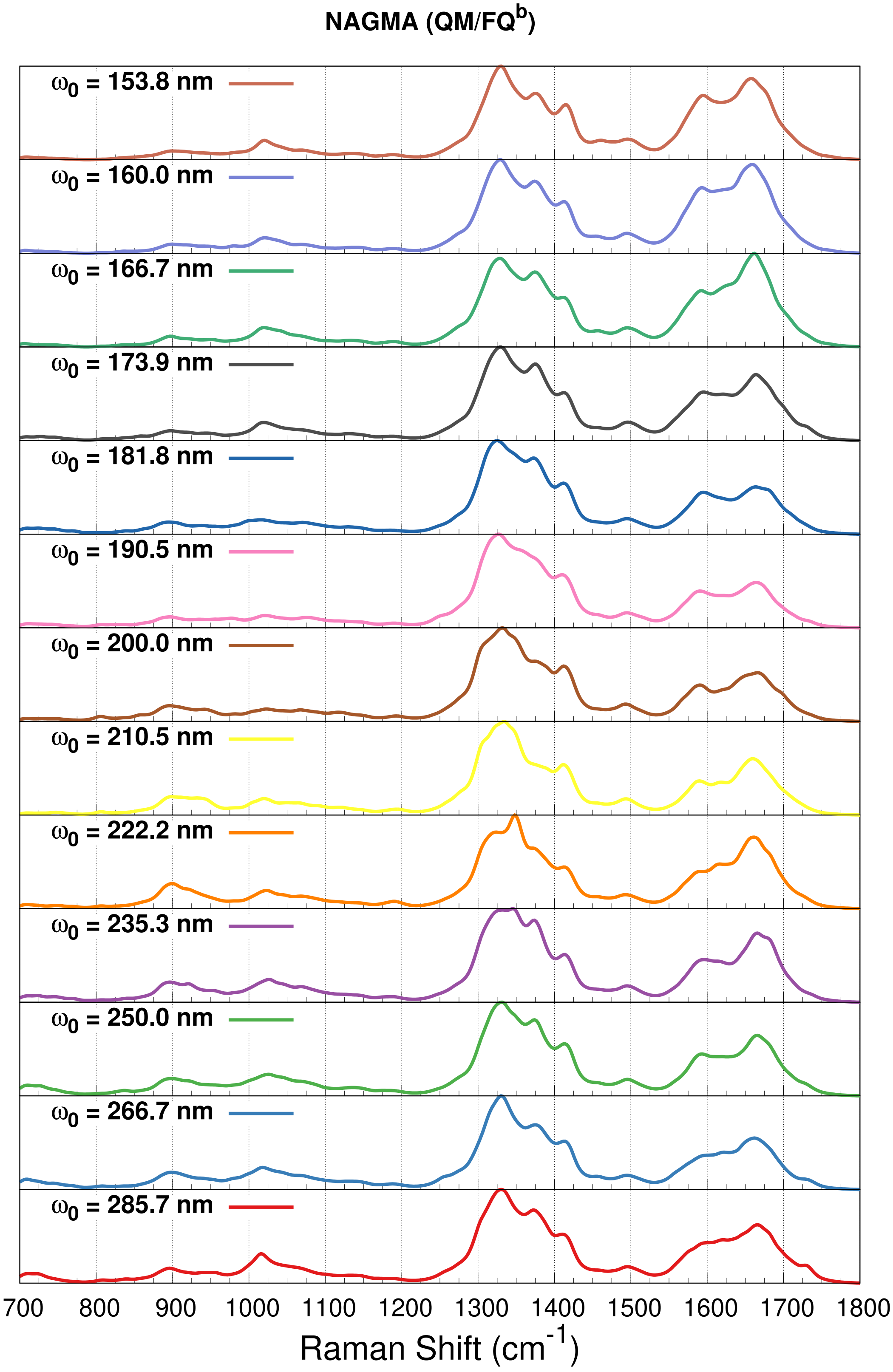

Figure S14: QM/FQ Resonance Raman Excitation profile of NAGMA in aqueous solution. FQ parameters were taken from Ref. 25

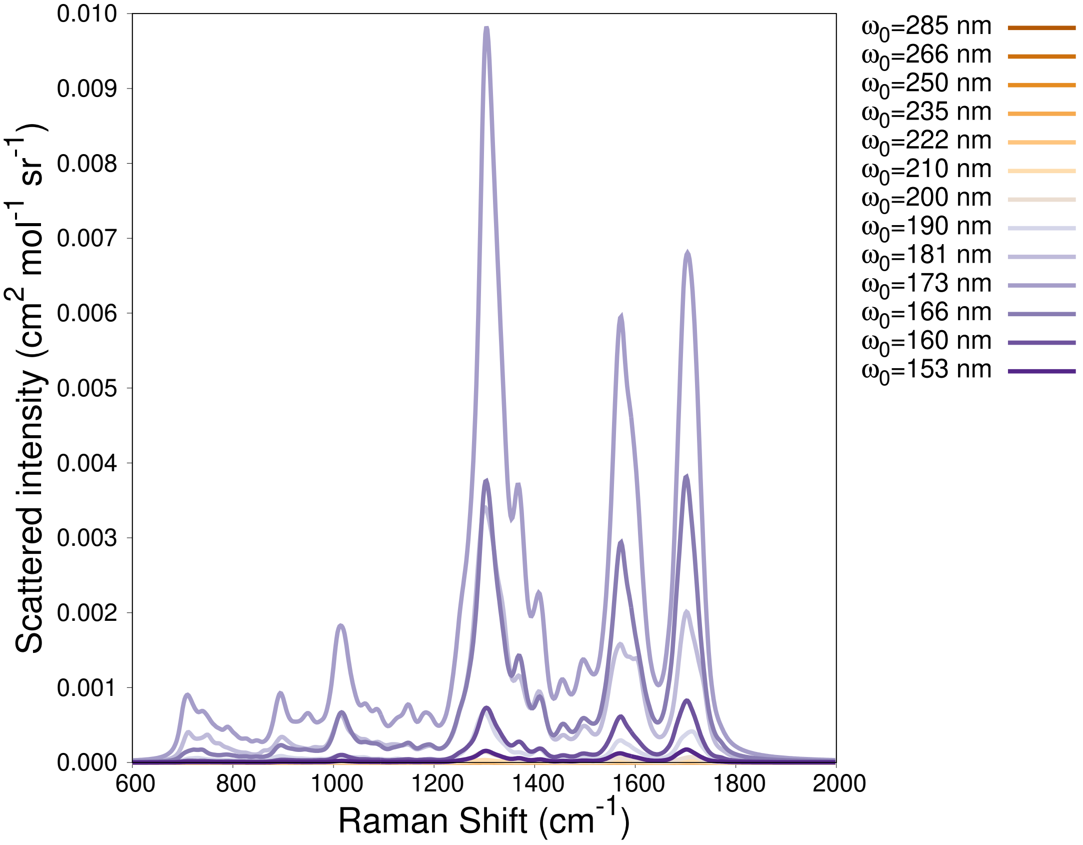

Figure S15: QM/TIP3P (Non-polarizable) Resonance Raman Excitation profile of NAGMA in aqueous solution.

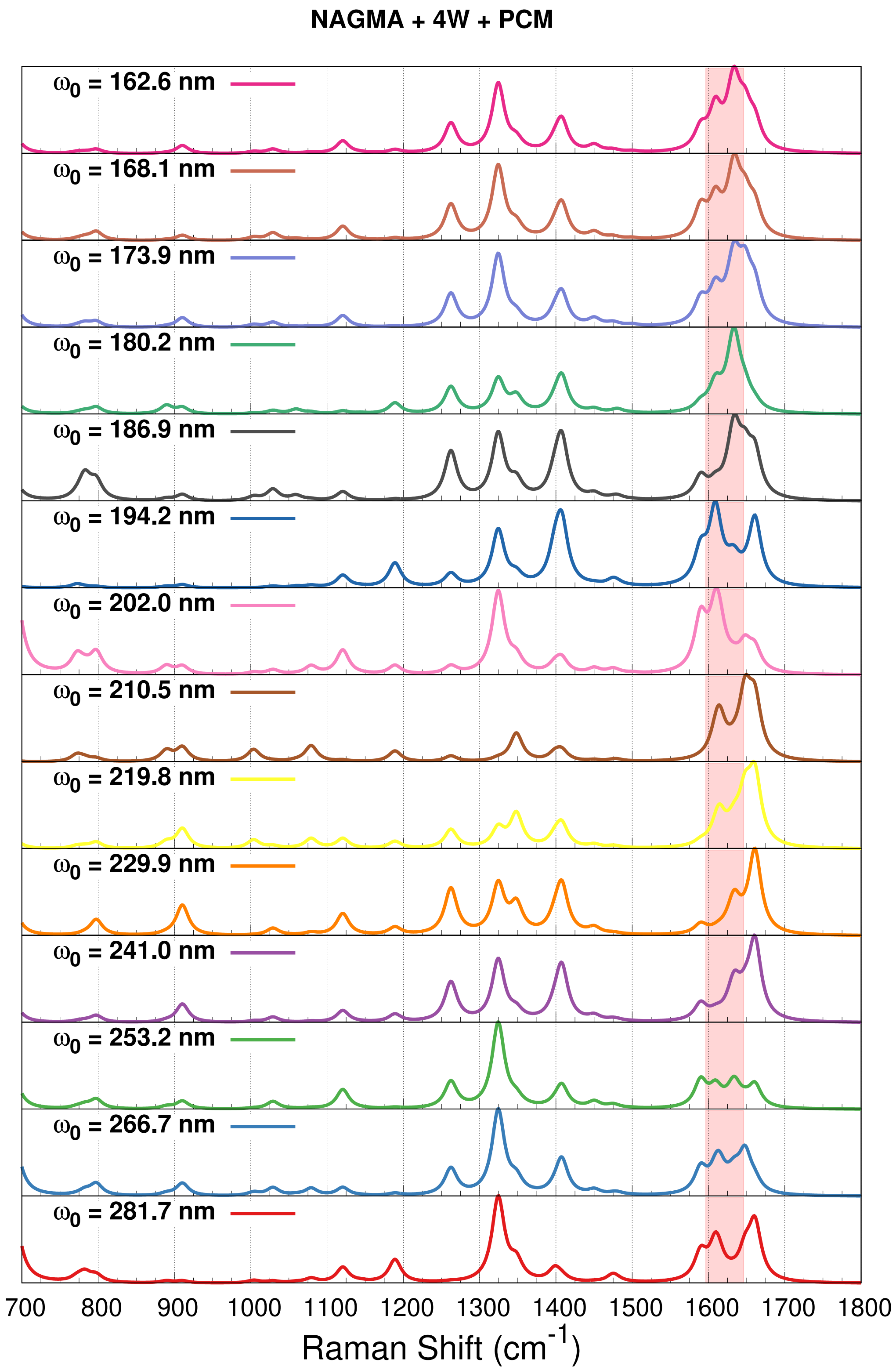

Figure S16: Resonance Raman spectra of the *supermolecule:* NAGMA + 4W in PCM at different excitation wavelengths. The region for the appearance of the AII signals is enclosed by an orange box.

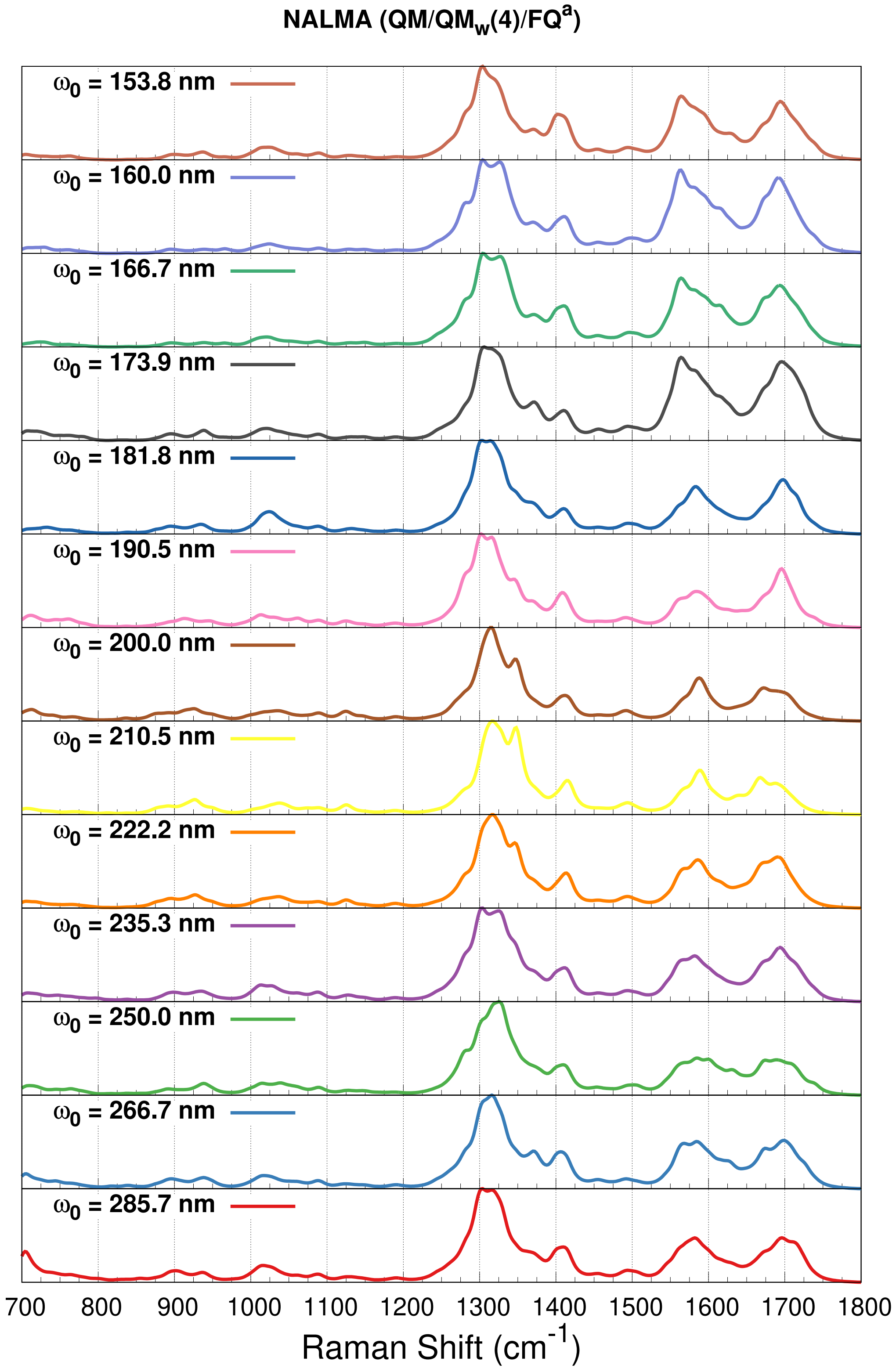

Figure S17: QM/QM$_w$/FQ Resonance Raman Excitation profile of NAGMA in aqueous solution when 4 water molecules were explicitly included in the QM portion. FQ parameters were taken from Ref. 24.

4.6.2 NALMA

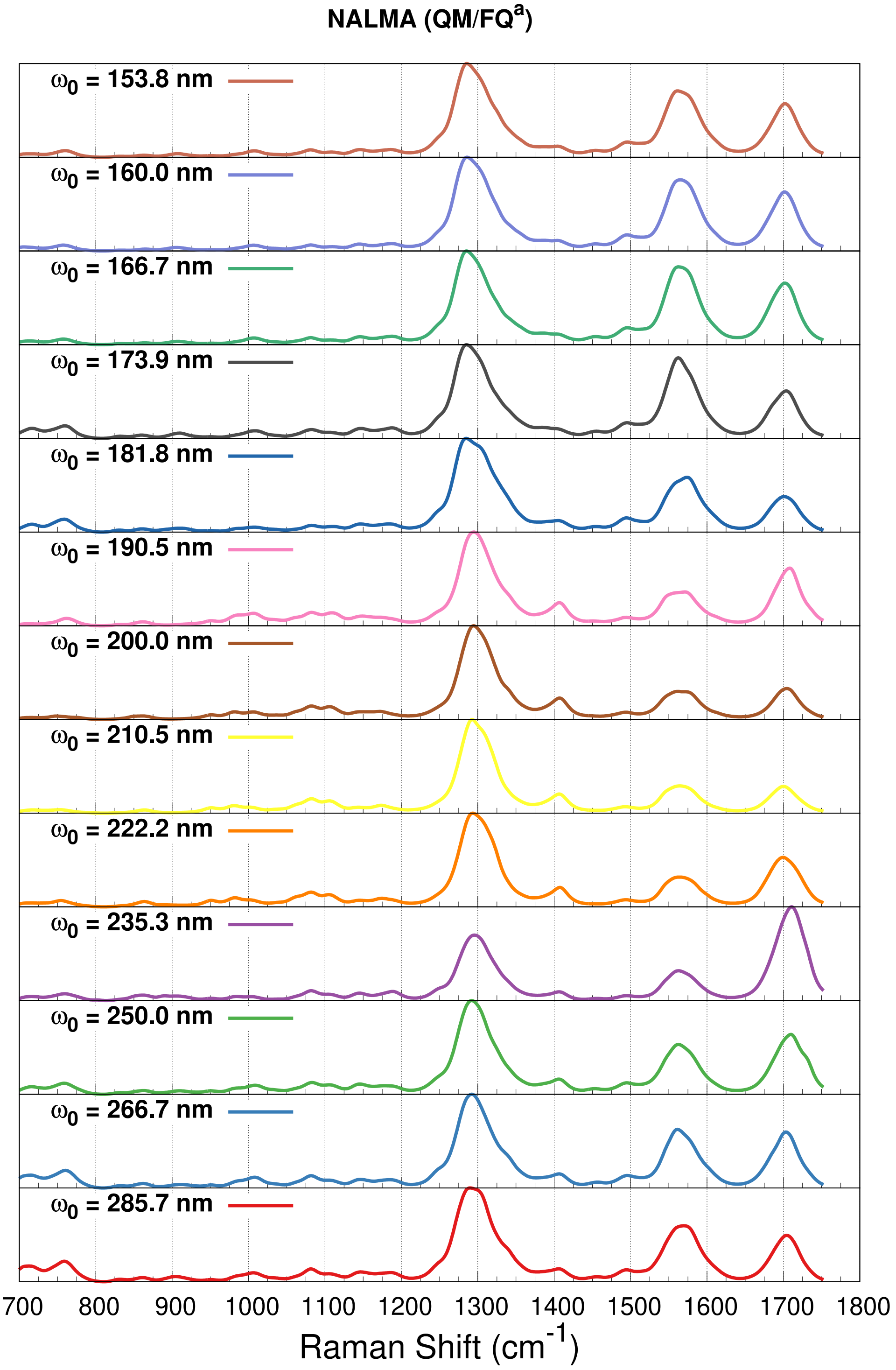

Figure S18: QM/FQ Resonance Raman Excitation profile of NALMA in aqueous solution. FQ parameters were taken from Ref. 24.

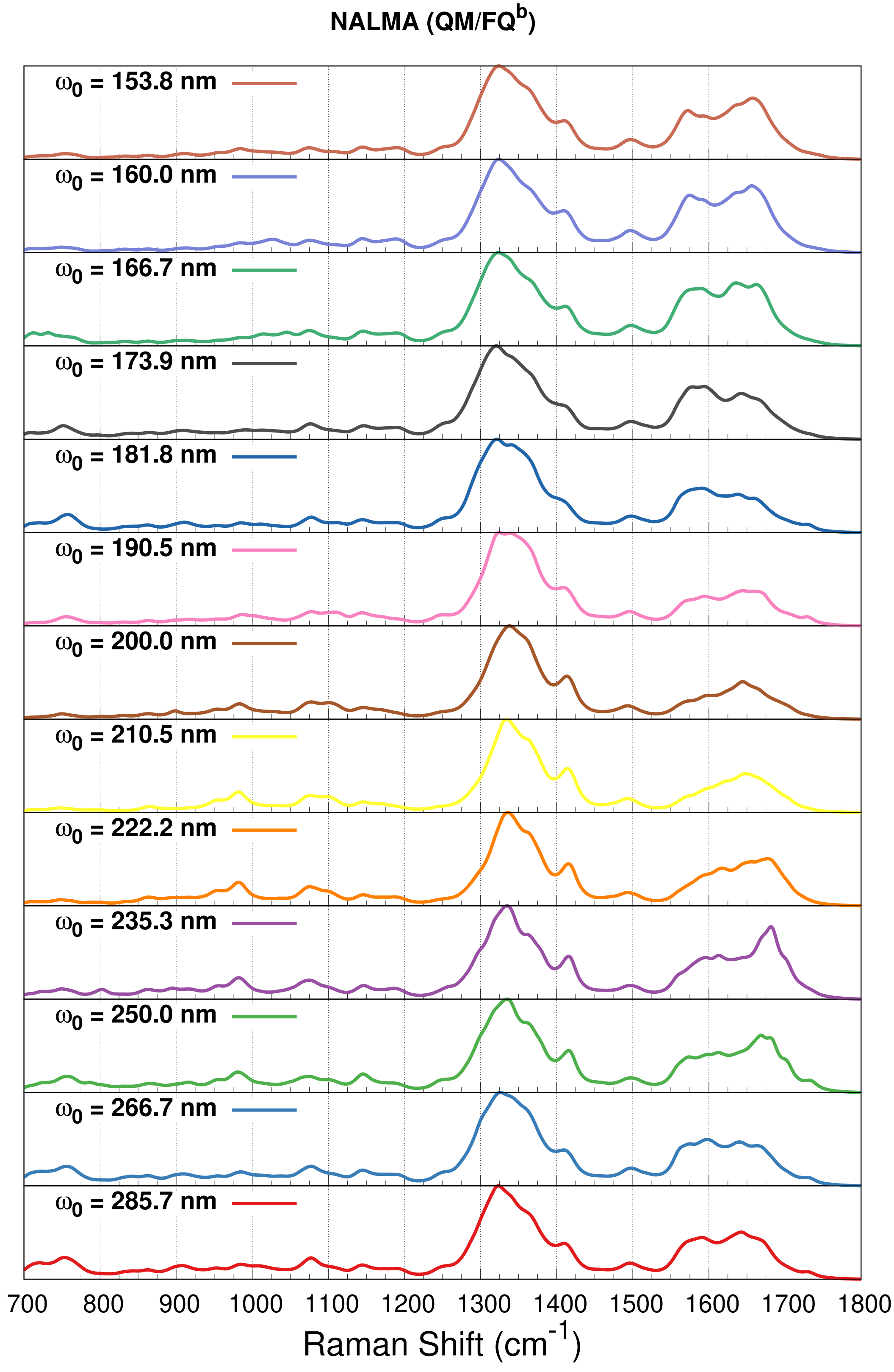

Figure S19: QM/FQ Resonance Raman Excitation profile of NALMA in aqueous solution. FQ parameters were taken from Ref. 25

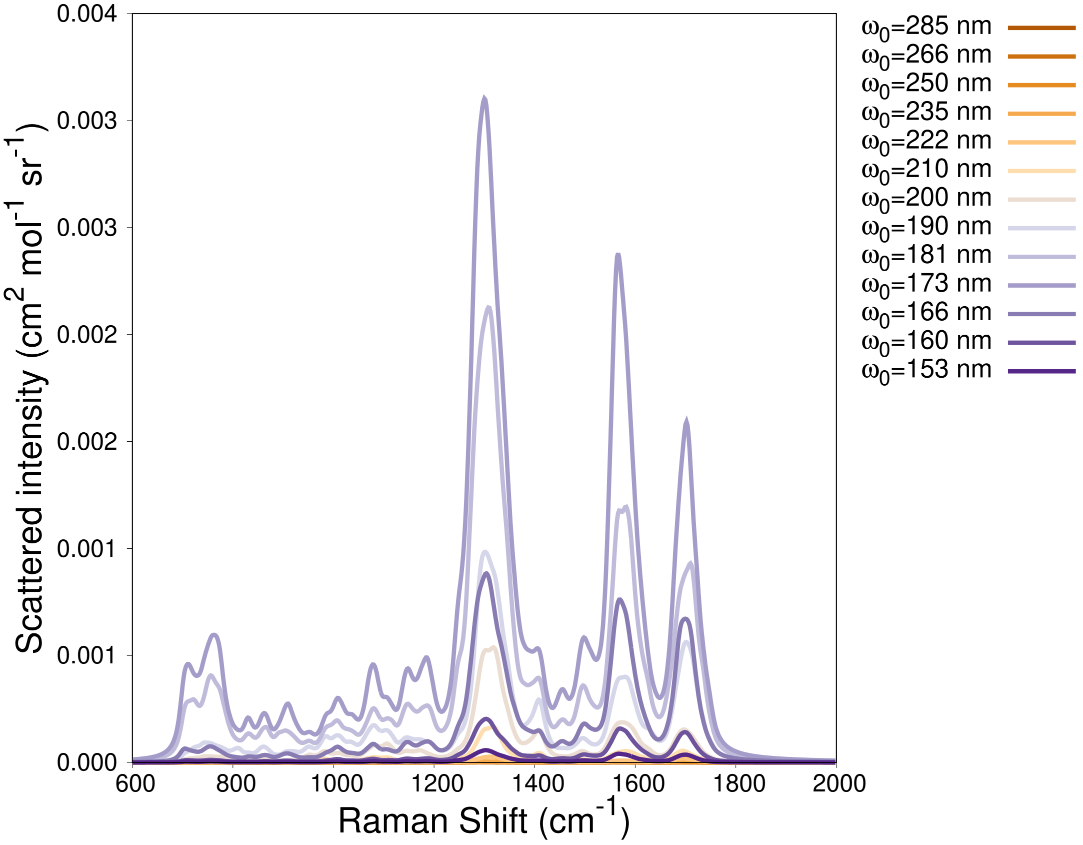

Figure S20: QM/TIP3P (Non-polarizable) Resonance Raman Excitation profile of NALMA in aqueous solution.

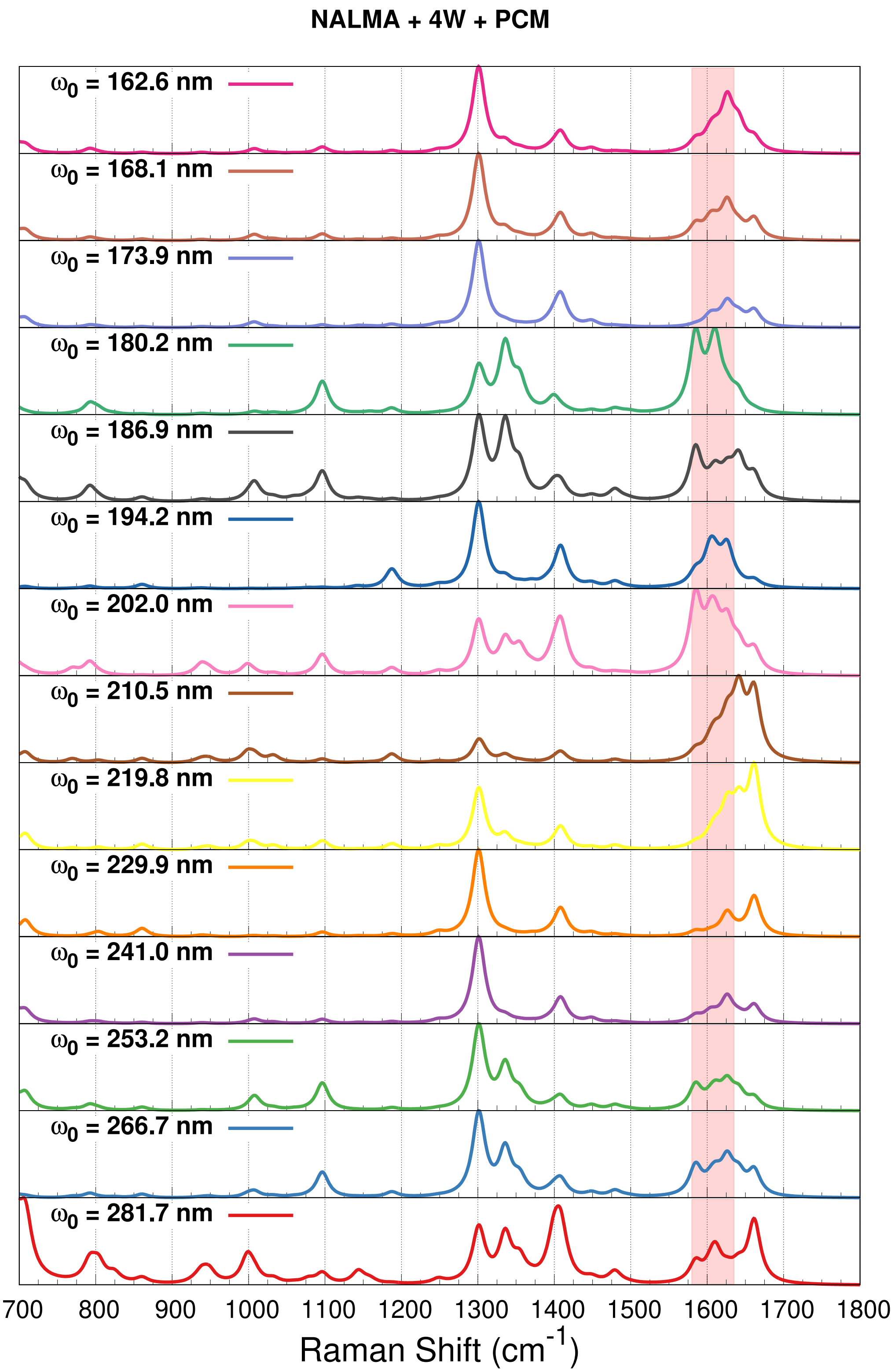

Figure S21: Resonance Raman spectra of the *supermolecule:* NALMA + 4W in PCM at different excitation wavelengths. The region for the appearance of the AII signals is enclosed by an orange box.

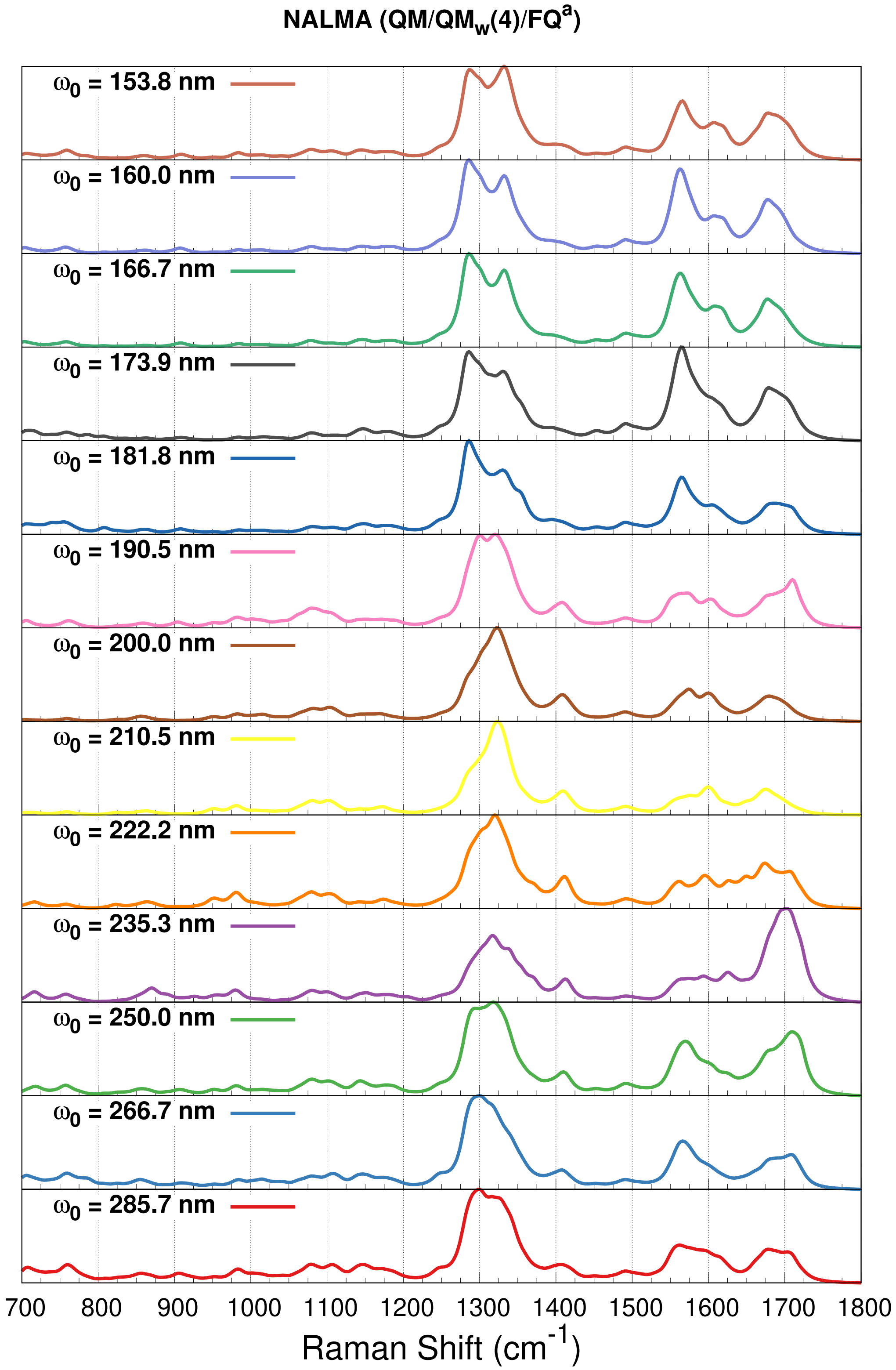

Figure S22: QM/QM$_w$/FQ Resonance Raman Excitation profile of NALMA in aqueous solution when 4 water molecules were explicitly included in the QM portion. FQ parameters were taken from Ref. 24.

4.7 Analysis of excitation wavelength and amide band intensities

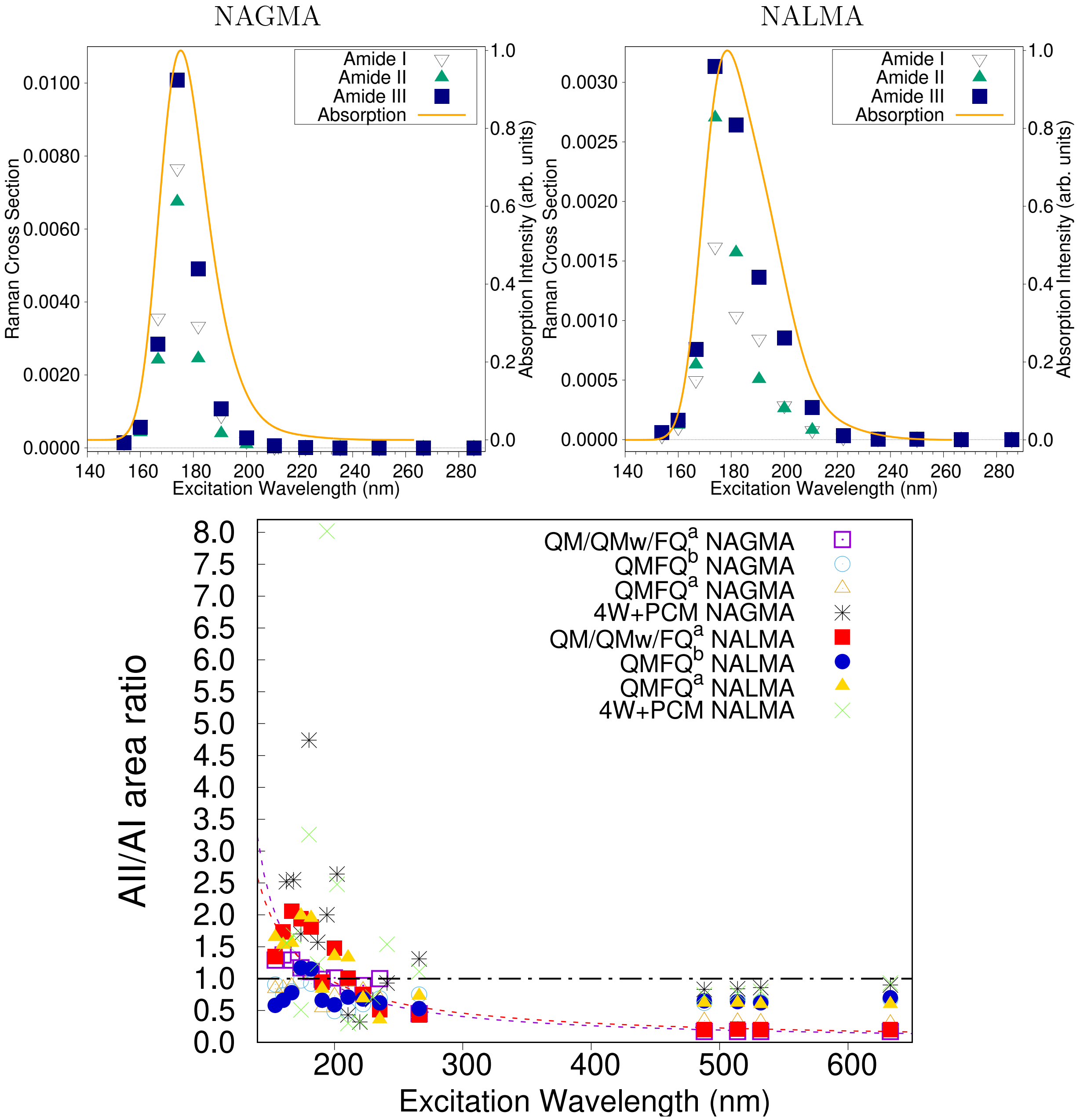

Figure S23: Excitation wavelength dependence of the Amide I, II, III bands intensity (top), and of Amide II/Amide I Raman area ratios (bottom). RR intensities (in $cm^2mol^{-1}sr^{-1}$) were calculated with a damping factor of 200 cm^{-1} and broadened using Lorentzian functions with a FWHM of 20 cm^{-1}. [a]FQ parametrization from Ref. 24, [b]FQ parametrization from Ref. 25

4.8 Details about the QM/QM$_w$/FQ approach

Table S5: Number of closest waters to the dipeptides, after fulfilling at least one of the following geometric criteria: $d_{\mathrm{H_w \cdots O=C}} < 2.5$ Å and $d_{\mathrm{O_w \cdots H-N}} < 2.5$ Å. For comparison purposes with the *supermolecule* structure, in the RR calculations we selected those snapshots having 4 water molecules close to the peptides.

n_W	NAGMA	NALMA
0	2	1
1	9	15
2	43	50
3	91	76
4	51	63
5	22	16
6	3	1
7	1	0

4.9 Experimental UVRR spectra of hydrated powders

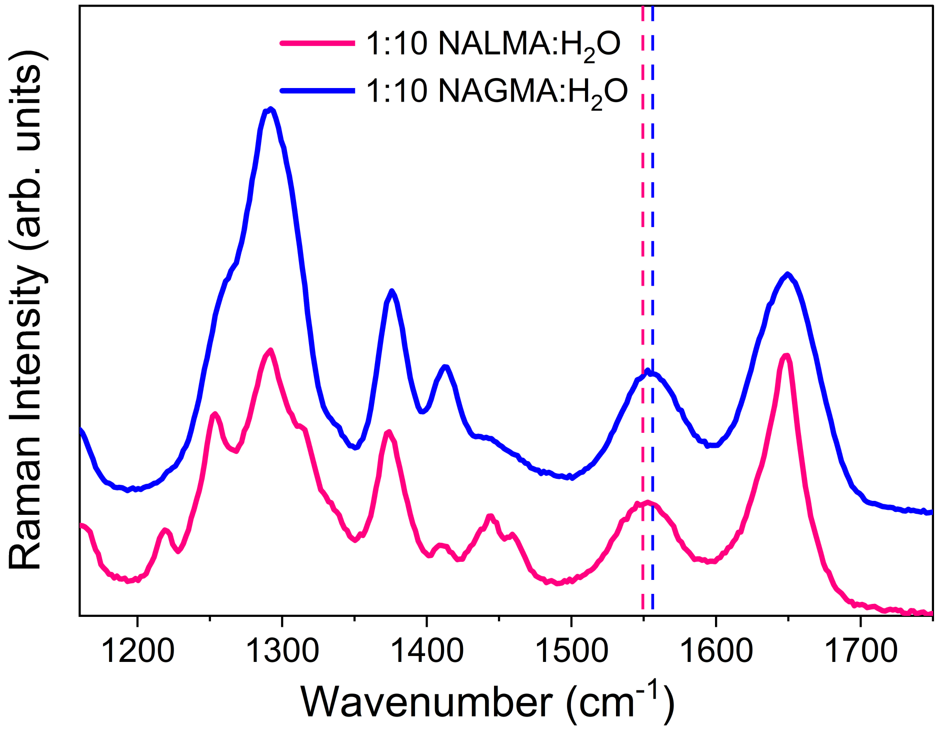

Figure S24: UVVR spectra of NAGMA and NALMA hydrated powders in the region of amides bands. The excitation wavelength was 266 nm and the measurements were carried out at room temperature. Vertical lines mark the position of the Amide II.

4.10 Simulated UVRR spectra in the gas phase for monomers and aggregates

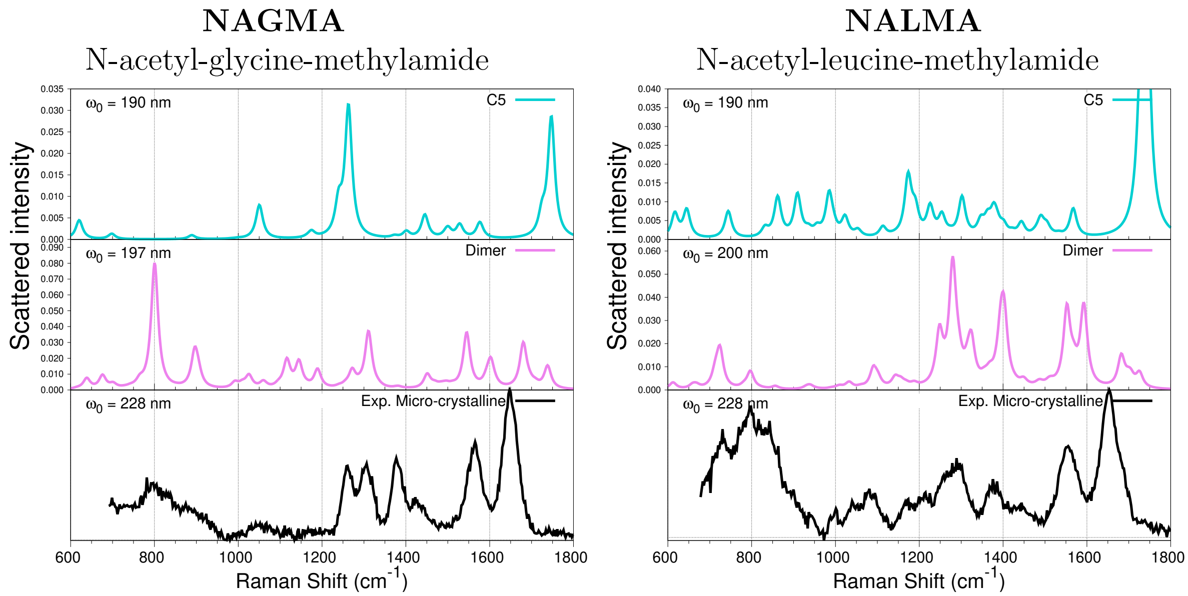

Figure S25: UV-resonance Raman spectra of NAGMA (left) and NALMA (right) in *vacuo*, calculated when their corresponding absorption maxima wavelengths are used to irradiate the system (see Table 1). *C5* is the lowest energy conformer of NAGMA and NALMA in the gas phase. RR intensities (in $cm^2mol^{-1}sr^{-1}$) were calculated with a damping factor of 200 cm^{-1} and broadened using Lorentzian functions with FWHM = 20 cm^{-1}. Experimental spectra were collected using 228 nm as excitation wavelength on micro-crystalline phases of the two dipeptides.

References

(1) Rossi, B.; Bottari, C.; Catalini, S.; D'Amico, F.; Gessini, A.; Masciovecchio, C. In *Molecular and Laser Spectroscopy*, Chapter 13 - Synchrotron-based Ultraviolet Resonance Raman Scattering for Material Science; Gupta, V., Ozaki, Y., Eds.; Elsevier, 2020; pp 447–482.

(2) Frisch, M. J.; Trucks, G. W.; Schlegel, H. B.; Scuseria, G. E.; Robb, M. A.; Cheeseman, J. R.; Scalmani, G.; Barone, V.; Petersson, G. A.; Nakatsuji, H. et al. Gaussian˜16 Revision B.01. 2016; Gaussian Inc. Wallingford CT.

(3) Perczel, A.; Angyan, J. G.; Kajtar, M.; Viviani, W.; Rivail, J. L.; Marcoccia, J. F.; Csizmadia, I. G. Peptide Models. 1. Topology of Selected Peptide Conformational Potential Energy Surfaces (Glycine and Alanine Derivatives). *J. Am. Chem. Soc.* **1991**, *113*, 6256–6265.

(4) Gould, I. R.; Cornell, W. D.; Hillier, I. H. A Quantum Mechanical Investigation of the Conformational Energetics of the Alanine and Glycine Dipeptides in the Gas Phase and in Aqueous Solution. *J. Am. Chem. Soc.* **1994**, *116*, 9250–9256.

(5) Bisetty, K.; Catalan, J. G.; Kruger, H. G.; Perez, J. J. Conformational Analysis of Small Peptides of the Type Ac–X–NHMe, where X= Gly, Ala, Aib and Cage. *J. Mol. Struct.: THEOCHEM* **2005**, *731*, 127–137.

(6) Masman, M. F.; Lovas, S.; Murphy, R. F.; Enriz, R. D.; Rodríguez, A. M. Conformational Preferences of N-Acetyl-l-leucine-N'-methylamide. Gas-Phase and Solution Calculations on the Model Dipeptide. *J. Phys. Chem. A* **2007**, *111*, 10682–10691.

(7) Boopathi, S.; Kolandaivel, P. Molecular Dynamics Simulations and Density Functional Theory Studies of NALMA and NAGMA Dipeptides. *J. Biomol. Struct. Dyn.* **2013**, *31*, 158–173.

(8) Leavitt, C. M.; Moore III, K. B.; Raston, P. L.; Agarwal, J.; Moody, G. H.; Shirley, C. C.; Schaefer III, H. F.; Douberly, G. E. Liquid Hot NAGMA Cooled to 0.4 K: Benchmark Thermochemistry of a Gas-Phase Peptide. *J. Phys. Chem. A* **2014**, *118*, 9692–9700.

(9) Cormanich, R. A.; Rittner, R.; Buehl, M. Conformational Preferences of Ac-Gly-NHMe in Solution. *RSC Advances* **2015**, *5*, 13052–13060.

(10) Weinhold, F.; Landis, C. R. *Discovering Chemistry with Natural Bond Orbitals*; Wiley-VCH, Hoboken NJ, 319pp, 2012.

(11) Weinhold, F.; Landis, C.; Glendening, E. What is NBO analysis and how is it useful? *International Reviews in Physical Chemistry* **2016**, *35*, 399–440.

(12) Glendening, E. D.; Badenhoop, J. K.; Reed, A. E.; Carpenter, J. E.; Bohmann, J. A.; Morales, C. M.; Karafiloglou, P.; Landis, C. R.; Weinhold, F. NBO 7.0. 2018; Theoretical Chemistry Institute, University of Wisconsin, Madison, WI.

(13) Abraham, M. J.; Murtola, T.; Schulz, R.; Páll, S.; Smith, J. C.; Hess, B.; Lindahl, E. GROMACS: High Performance Molecular Simulations Through Multi-Level Parallelism from Laptops to Supercomputers. *SoftwareX* **2015**, *1-2*, 19 – 25.

(14) Wang, J.; Wolf, R. M.; Caldwell, J. W.; Kollman, P. A.; Case, D. A. Development and Testing of a General Amber Force Field. *J. Comput. Chem.* **2004**, *25*, 1157–1174.

(15) Marenich, A. V.; Jerome, S. V.; Cramer, C. J.; Truhlar, D. G. Charge Model 5: An Extension of Hirshfeld Population Analysis for the Accurate Description of Molecular Interactions in Gaseous and Condensed Phases. *J. Chem. Theory Comput.* **2012**, *8*, 527–541.

(16) Mark, P.; Nilsson, L. Structure and Dynamics of the TIP3P, SPC, and SPC/E Water Models at 298 K. *J. Phys. Chem. B* **2001**, *105*, 9954–9960.

(17) Darden, T.; York, D.; Pedersen, L. Particle Mesh Ewald: An N·log(N) Method for Ewald Sums in Large Systems. *J. Chem. Phys.* **1993**, *98*, 10089–10092.

(18) Berendsen, H. J.; Van Gunsteren, W. F. Practical Algorithms for Dynamic Simulations. *Molecular-dynamics simulation of statistical-mechanical systems* **1986**, 43–65.

(19) Parrinello, M.; Rahman, A. Strain Fluctuations and Elastic Constants. *J. Chem. Phys.* **1982**, *76*, 2662–2666.

(20) Brehm, M.; Kirchner, B. TRAVIS - A Free Analyzer and Visualizer for Monte Carlo and Molecular Dynamics Trajectories. *Journal of Chemical Information and Modeling* **2011**, *51*, 2007–2023.

(21) Brehm, M.; Thomas, M.; Gehrke, S.; Kirchner, B. TRAVIS—A Free Analyzer for Trajectories from Molecular Simulation. *J. Chem. Phys.* **2020**, *152*, 164105.

(22) Daura, X.; Gademann, K.; Jaun, B.; Seebach, D.; van Gunsteren, W. F.; Mark, A. E. Peptide Folding: When Simulation Meets Experiment. *Angewandte Chemie International Edition* **1999**, *38*, 236–240.

(23) Giovannini, T.; Egidi, F.; Cappelli, C. Molecular Spectroscopy of Aqueous Solutions: A Theoretical Perspective. *Chem. Soc. Rev.* **2020**, *49*, 5664–5677.

(24) Rick, S. W.; Stuart, S. J.; Berne, B. J. Dynamical Fluctuating Charge Force Fields: Application to Liquid Water. *J. Chem. Phys.* **1994**, *101*, 6141–6156.

(25) Giovannini, T.; Lafiosca, P.; Chandramouli, B.; Barone, V.; Cappelli, C. Effective yet Reliable Computation of Hyperfine Coupling Constants in Solution by a QM/MM Approach: Interplay between Electrostatics and Non-Electrostatic Effects. *J. Chem. Phys.* **2019**, *150*, 124102.

(26) Santoro, F.; Cappelli, C.; Barone, V. Effective Time-Independent Calculations of Vibrational Resonance Raman Spectra of Isolated and Solvated Molecules Including Duschinsky and Herzberg–Teller Effects. *J. Chem. Theory Comput.* **2011**, *7*, 1824–1839.

(27) Long, D. A. *The Raman Effect*, Vibrational Raman Scattering; John Wiley & Sons, Ltd, 2002; Chapter 5, pp 85–152.

(28) Long, D. A. *The Raman Effect*, The Polarizability Tensor; John Wiley & Sons, Ltd, 2002; Chapter 24, pp 471–495.
